# Pathogenomic analysis reveals clinically relevant epithelial-mesenchymal plasticity in esophageal squamous cell carcinoma

**DOI:** 10.7150/thno.125381

**Published:** 2026-01-14

**Authors:** Ruzhen Chen, Chenyi Xie, Ziyu Ning, Meng Yang, Zezhuo Su, Jiahui Chen, Kunheng Du, Yihuai Hu, Chu Han, Shaojun Zhang, Qingling Zhang, Meng Liu, Zaiyi Liu

**Affiliations:** 1 School of Medicine, South China University of Technology, Guangzhou 510006, China.; 2 Department of Radiology, Guangdong Provincial People's Hospital (Guangdong Academy of Medical Sciences), Southern Medical University, Guangzhou 510080, China.; 3 Affiliated Tumor Hospital of Xinjiang Medical University, Urumqi 830011, Xinjiang, China.; 4 Department of Orthopaedics and Traumatology, School of Clinical Medicine, Li Ka Shing Faculty of Medicine, The University of Hong Kong, Pok Fu Lam, Hong Kong SAR, China.; 5 Guangdong Provincial Key Laboratory of Artificial Intelligence in Medical Image Analysis and Application, Guangzhou 510080, China.; 6 Department of Pathology, Guangdong Provincial People's Hospital (Guangdong Academy of Medical Sciences), Southern Medical University, Guangzhou 510080, China.; 7 Department of Thoracic Surgery, Guangdong Provincial People's Hospital (Guangdong Academy of Medical Sciences), Southern Medical University, Guangzhou 510080, China.; 8 Guangdong Academy of Medical Sciences and Medical Research Institute, Guangdong Provincial People's Hospital (Guangdong Academy of Medical Sciences), Southern Medical University, Guangzhou 510080, China.

**Keywords:** esophageal squamous carcinoma, epithelial mesenchymal transition, histopathology, bulk sequencing, single-cell sequencing, spatial transcriptomes, deep learning

## Abstract

**Rationale**: Esophageal squamous cell carcinoma (ESCC) is a highly aggressive malignancy. The metastasis and poor prognosis of ESCC are closely associated with tumor microenvironment (TME) heterogeneity, which is driven by epithelial-mesenchymal transition (EMT). Clinically, how to diagnose and target EMT progression remains a key challenge for ESCC.

**Methods**: Integration of pathological images and bulk RNA sequencing profiles identified a high-risk subtype exhibiting EMT enrichment and immunosuppression. Single-cell and spatial transcriptomics revealed EMT macrostates and their spatial distribution. The role of *CACNA1C* in programming malignant phenotype was tested *in vitro*. A pathological image-based deep learning model successfully predicted the spatial expression distribution of *CACNA1C*, indicating possible clinical utility.

**Results**: EMT progression comprised three macrostates: the early state (high epithelial and metastatic potential), the stable state (hybrid E/M phenotype and high stemness), and the late state (high mesenchymal and invasive propensity). *ITGA3* and *ITGB4* antagonistically regulate malignant phenotype in the early state. Notably, suppression of *CACNA1C* induced transdifferentiation from stable/late-state cells to normal epithelium-like cells.

**Conclusions**: This study provides novel insights into the EMT mechanism in ESCC, proposes an intervention strategy, and emphasizes the promising clinical application of pathological images in EMT assessment.

## Introduction

Esophageal cancer is the seventh leading cause of cancer-related death worldwide 1. Esophageal squamous cell carcinoma (ESCC) accounts for approximately 90% of esophageal cancer cases, and more than 70% of ESCC cases are locally advanced at the time of diagnosis 2. ESCC is characterized by aggressive submucosal spread and lymphatic metastasis, and significantly poorer survival outcomes are observed in patients with metastatic disease 3. While surgical resection is the fundamental treatment for ESCC 4, emerging therapeutic targets and molecular classifications derived from multiomics technologies have been increasingly proposed to guide personalized therapy 5. Understanding the ESCC tumor microenvironment (TME) is essential for overcoming treatment resistance, refining subtype-specific treatment strategies, and improving patient outcomes.

The degree of epithelial-to-mesenchymal transition (EMT) has been reported to be a predictor of tumor invasion and lymph node metastasis 6, 7. EMT is a cellular process in which polarized epithelial cells undergo extensive molecular reprogramming and phenotypic transition 8. EMT occurs not only during embryonic development, tissue regeneration, and wound healing but also in tumors 9, which leads to a reduction in adhesion between cells and an increase in the ability to migrate, thus initiating the transformation from a benign tumor to an invasive tumor. Recent studies have shown that EMT encompasses a continuum of phenotypes, with multiple mixed states driving the transition from a complete epithelial phenotype to a complete mesenchymal phenotype 10, 11. Elucidation of EMT differentiation trajectories is crucial for understanding ESCC invasiveness and metastasis and had significant potential to inform targeted therapies and improve patient survival; however, the specific EMT intermediate states and their functions remain incompletely defined.

Tumor morphology and architecture reflect biologically relevant subtypes, and integrating spatial pathology with genomic data enables deeper insights into the TME and biomarker development 12. For example, Wang *et al.* and Yoo *et al.* performed unsupervised clustering on quantitative features characterizing the cell nuclei and the spatial relationships between cancer cells and lymphocytes to develop histological subtypes in liver cancer and colorectal cancer, respectively 13, 14. Zhao *et al.* further incorporated stromal cell features and identified clinically relevant subtypes of breast cancer 15. Recent deep learning advances have enabled cost-effective, reliable biological stratification directly from histopathological images in both clinical workflows and retrospective cohorts, revealing novel insights into tumor heterogeneity 16, 17. The integration of pathogenomics and deep learning for multimodal biomarker discovery enables the characterization of tumor heterogeneity and clarification of previously unexplored genomic mechanisms in ESCC histological subtypes, advancing both oncology research and clinical decision-making.

In this study, we identified two pathological subtypes of ESCC with prognostic differences. We performed integrated analysis of genomics data for paired samples to comprehensively characterize the molecular dynamics and TME landscapes underlying individual subtypes, identified EMT as a critical determinant of prognosis, and mined therapeutic targets and functional ligand-receptor pairs for the high-risk subtype. In addition, we constructed ESCC-specific EMT trajectories and identified three macrostates with distinct functions. We found that *ITGA3* and *ITGB4* are functionally mutually exclusive and that *CACNA1C* is a promising trans-differentiation target for EMT. Finally, we developed a deep learning model to predict the expression and spatial distribution of *CACNA1C*. In conclusion, our study reveals the widespread impact of EMT on ESCC invasion, metastasis, prognosis, and treatment.

## Methods

### Multi-center data collection

This study has been approved by the institutional review boards at each hospital and informed consent was waived due to retrospective nature. This retrospective study involved a discovery cohort (Affiliated Tumor Hospital of Xinjiang Medical University, XJ, n = 125), as well as a validation cohort (Guangdong Provincial People's Hospital, GDPH, n = 78). Eligible participants met the following criteria: (i) a confirmed pathological diagnosis of ESCC; (ii) receipt of primary curative-intent surgical resection; and (iii) availability of comprehensive histological data. Patients were excluded if they had: (i) undergone any preoperative anti-cancer treatment; (ii) a prior history of malignancy or coexisting cancers; or (iii) missing or incomplete clinical, imaging, or pathological records. The overall survival (OS) as the primary endpoint, defined as the time interval from surgical resection to death from any cause. The minimum follow-up period was 36 months after surgery. We collected clinicopathologic characteristics from medical records and assessed followed-up information by pathology/imaging reports or telephone follow-up. Baseline characteristics were collected, including age, gender, tumor location, stage, T stage, N stage, and adjuvant therapy record. Statistical comparisons across cohorts were calculated by Chi-squared test (Table S1).

### Public data collection

The published clinical specimens here were collected from the TCGA Research Network (https://portal.gdc.cancer.gov/) and the Gene Expression Omnibus (https://www.ncbi.nlm.nih.gov/geo/). From the GDC Data Portal, we identified esophageal squamous cell carcinoma (ESCC) samples using the following selection criteria: PrimarySite = "Esophagus", DiseaseType = "Squamous cell neoplasms", SampleType = "Primary Tumor", and TreatmentOrTherapy = "No". We further refined our selection to include only cases with available H&E-stained diagnostic slides from surgical specimens. We employed the GDC Data Transfer Tool for download of clinical information and histopathological slides, while utilizing the TCGAbiolinks R package 18 to retrieve single nucleotide variation (SNV) data, copy number variation (CNV) data, and transcriptomic profiles. Additionally, we acquired single-cell RNA sequencing data (GSE160269) of ESCC clinical specimens from the GEO database.

Cell line data were obtained from multiple public resources: transcriptomic profiles from the Cancer Cell Line Encyclopedia (CCLE) 19 (https://sites.broadinstitute.org/ccle/), drug response data from the Genomics of Drug Sensitivity in Cancer (GDSC) 20 (https://www.cancerrxgene.org/), metastatic potential data from MetMap500 21 (https://depmap.org/metmap/), genetic perturbation data from the Library of Integrated Network-based Cellular Signatures (LINCs) 22 (https://lincsproject.org/), and genetic dependency data from DepMap 23 (https://depmap.org/portal/data_page/).

### H&E-stained section scanning

The formalin-fixed and paraffin-embedded tissue sections were collected by surgical resection and stained with H&E. A representative section of each patient was selected by experienced pathologists (Z.N. and Q.Z.) blinding of patient outcomes. The H&E-stained sections were scanned and digitalized using whole-slide scanners at 400×magnification with a resolution of 0.25 μm per pixel. All WSIs were manually checked for quality control, excluding images with blurry areas, and light- or over-stained areas.

### Spatial transcriptomic sequencing

For 10x Visium v2 experiment, FFPE tissues were sectioned (5 μm), and following RNA quality assessment, sections underwent deparaffinization, H&E staining, and imaging. Probe hybridization employed the RTL technique, and ligation products were captured on Visium slides via poly(dT) sequences. Subsequent library construction included mRNA digestion, probe extension for barcode/UMI incorporation, alkaline elution, pre-amplification, indexing, and cleanup. Final libraries were sequenced on an Illumina platform.

For 10x Visium HD experiment, fresh tissue was fixed, paraffin-embedded, and sectioned. After deparaffinization, H&E staining, imaging, and decrosslinking per the manufacturer's protocol, probe hybridization and library construction were performed using the Visium HD kit. Libraries were sequenced in PE-150 mode on an Illumina platform.

For Stereo-seq experiment, fresh tissue was OCT-embedded, frozen, and cryosectioned (10 μm). Sections were adhered to a Stereo-seq poly-T chip, then processed for methanol fixation, H&E staining, imaging, permeabilization, reverse transcription, and cDNA purification according to kit instructions. Libraries were prepared and sequenced on a DNBSEQ-T7 instrument.

For 10x Xenium experiment, FFPE sections (5 μm) were mounted on Xenium slides. After histopathological assessment, spatial profiling was conducted using the Xenium platform with a 5K gene panel. The workflow included deparaffinization, probe hybridization, ligation, rolling circle amplification. Image fields of view were computationally stitched using the DAPI channel to reconstruct whole-tissue spatial maps. Cell segmentation was performed using a multimodal approach integrating nuclear and cytoplasmic signals, and detected transcripts were assigned to individual cells based on their spatial localization. Following completion of imaging, slides were subjected to post-run H&E staining and whole-slide scanning. H&E images were aligned with Xenium morphology images using Xenium Explorer software to enable visualization of spatial gene expression within the histological context.

### Cell culture and transfection

Human ESCC cell line TE-8 (Ethephon, YCL-0533) was selected because of intrinsic *CACNA1C* expression allowing for knock-down of function assay. TE-8 was cultured in RPMI1640 media supplemented with 10% FBS and 1% P/S under 5% CO_2_ conditions. Short tandem repeat profiling of the cell lines was performed to ensure integrity of cell line.

For knock-down assay, TE-8 was transfected with siRNA targeting *CACNA1C* or control siRNA (Hycyte, HX-H-S3-16863). At 48 hours post-transfection, RT-qPCR was performed to confirm that *CACNA1C* expression was decreased.

### Cellular invasion assay

For cell invasion assay, cell lines were plated in a 24-well plate at 9.0 × 10^4^ cells per well. Invasion was evaluated using the Matrigel 24-well Cell Invasion Chamber (8 μm, Corning, 354480). After incubation at 37 °C with 5% CO₂ for 24 h, non-invasion cells on the upper membrane surface were removed by gentle scraping with a cotton swab. Membranes were then fixed in 4% formaldehyde (15 min, room temperature) and stained with 0.1% crystal violet (20 min). Invasive cells on the lower membrane surface were visualized under bright-field microscopy. Five different 200× filed images were taken for each well, and the number of invasive cells was counted by ImageJ (National Institutes of Health).

### Cellular proliferation and colony formation assay

For cell proliferation assay, cell lines were plated in a 96-well plate at 1.0 × 10^3^ cells per well. Proliferation was evaluated using the CellTiter-Glo Luminescent Cell Viability Assay (Promega, G7570). After incubation at 37 °C with 5% CO₂ for 48 h, media was replaced to 200 μL of media containing 50% CellTiter-Glo reagent. Contents were mixed on an orbital shaker for 2 min, and incubated at room temperature for 10 min. The intensity of luminescent signal was detected by Luminometer.

For cell colony formation assay, cell lines were plated in triplicate in a 6-well culture plates at 1.0 × 10^3^ cells per well. Seven days after plating, cells were fixed and stained with crystal violet. The colony accounts were counted by ImageJ (National Institutes of Health).

### Pathological feature extraction and analysis

The analysis pipeline began by segmenting whole-slide images (WSIs) into 4096 * 4096-pixel patches representing TME. Each patch was processed at 40× magnification using Hovernet 24, a pre-trained cell segmentation model on Panuke dataset 25, to identify tumor cells, immune cells, and stromal cells. Spatial positions of these cell types were extracted and used to construct topological graphs via minimum spanning trees. From these graphs, we derived six distinct cellular interaction subnetworks: tumor-tumor (T-T), tumor-lymphocyte (T-I), tumor-stroma (T-S), lymphocyte-lymphocyte (I-I), lymphocyte-stroma (I-S), and stroma-stroma (S-S). Patch-level features were aggregated into WSI-level representations using four statistical measures (mean, variance, skewness, and kurtosis), generating a final pathological feature matrix of 1,008 dimensions per WSI.

To evaluate cross-cohort feature consistency, we first applied the ComBat function from the sva R package 26 for batch effect correction, followed by PCA dimensionality reduction for visualization using the first two principal components. Intra-cohort heterogeneity was assessed by selecting the top 10 principal components contributing most to cohort variation (determined by the elbow method) and performing K-means clustering to stratify cases into two pathological subtypes. The clinical relevance of these phenotypes was evaluated through Kaplan-Meier survival analysis with log-rank tests, supplemented by Cox proportional hazards modeling to quantify survival impact, using *p* < 0.05 as the significance threshold.

### Bulk transcriptomic feature extraction and functional profiling

Using the low-risk subtype as reference, we identified differentially expressed genes (DEGs) between pathological subtypes using the DESeq2 R package 27, obtaining log2 fold-change (logFC), *p*-values for each gene. BH method was applied for *p*-value significance correction. We performed gene set over representation analysis (ORA) on GO (Gene Ontology) terms from MSigDB, using significantly upregulated genes as the input gene list, implemented using the ClusterProfiler R package 28. We conducted variation and enrichment analyses on gene sets from MSigDB, including Hallmark, KEGG, Reactome, CGP, and PID collections, implemented using the GSVA 29 and clusterProfiler R packages, respectively.

### Pathology-pathway association

We employed regularized generalized linear models to investigate associations between pathological features and molecular pathways. Specifically, the model incorporated 1,008 WSI-level pathological features as predictors and pathway GSVA scores as response variables, implemented using the glmnet R package 30. Model performance was evaluated via prediction accuracy in five-fold cross-validation, with lambda (regularization parameter) selection optimized through nested ten-fold cross-validation within each fold. Finally, we assessed model accuracy by computing Spearman's rank correlation coefficients between predicted pathway scores and actual GSVA scores for each pathway.

### Disease-drug-target association

We computed drug-disease associations using the summarized Reverse Gene Expression Score (sRGES) established by Chen et al. 31 To validate the association reliability, we correlated sRGES with drug AUC values from cell-line experiments. For target prioritization, we retrieved protein targets of drugs from chEMBL, and performed target enrichment analysis on the sRGES-ranked drug list. The enrichment score was calculated using the single sample gene set enrich analysis (ssGSEA), and its significance was computed by a permutation test.

### Microenvironment deconvolution

We performed cell type deconvolution of bulk RNA-seq data using both CIBERSORTx 32 (estimating 22 cell types) and xCell 33 (estimating 64 cell types), implemented using the R package immunedeconv 32. Additionally, we inferred receptor-ligand interaction activity from bulk transcriptomes using the absolute model of BulkSignalR R package 34, followed by univariate Cox regression to evaluate the prognostic significance of each receptor-ligand pair. Corrected *p*-values were calculated with the BH method.

### Single-cell transcriptomic preprocessing

We retrieved a ESCC single-cell RNA sequencing atlas from GEO and processed it into Seurat object using the Seurat R package 35 for downstream analysis. Cell subtype marker genes were retrieved from corresponding paper.

For quality control, we retained cells with ≥200 features and <10% mitochondrial gene, while removing genes expressed in <0.1% of cells along with all mitochondrial and ribosomal genes, followed by doublet detection and removal using the DoubletFinder R package 36. Count matrices were log-normalized (scale factor = 10,000) via the NormalizeData function, and the top 2,000 highly variable genes (HVGs) were identified using FindVariableFeatures function with the vst method. HVG expression matrices were then scaled and centered using ScaleData. Dimensionality reduction was performed via PCA with optimal PC numbers determined by the ElbowPlot method, followed by batch effect correction using Harmony. These selected PCs were subsequently employed for cell clustering, constructing sNN graphs (FindNeighbors) followed by Louvain algorithm-based clustering (FindClusters) and UMAP visualization.

For cell annotation, we first assigned major cell types based on expression of canonical marker genes. Subsequently, using cell subtype markers provided by Zhang et al., we computed AUC scores for each subtype within major cell categories via the AUCell R package 37, ultimately assigning each cell its optimal subtype based on maximum AUC values.

We quantified gene module activity for each cell using the AddModuleScore function. Epithelial scores were computed as the average expression of canonical epithelial markers (*KRT14, KRT17, KRT6A, KRT5, KRT19, KRT8, KRT16, KRT18, KRT6B, KRT15, KRT6C, KRTCAP3, SFN, EPCAM*), while mesenchymal scores represented the mean expression of established mesenchymal markers (*VIM, CDH2, FOXC2, SNAI1, SNAI2, TWIST1, FN1, ITGB6, MMP2, MMP3, MMP9, SOX10, GSC, ZEB1, ZEB2, TWIST2*). The epithelial-mesenchymal transition (EMT) score was derived by subtracting epithelial scores from mesenchymal scores. Proliferation scores were calculated based on the average expression of S-phase and G2M-phase genes from the cc.genes function. Additionally, we assessed the differentiation potential using the CytoTRACE2 R package 38.

### Cell trajectory inference

Monocle2 R package 39 was used to perform cell trajectory inference. Monocle2 generates the trajectory using the principal graph algorithm. The top 50 highly expressed genes were selected for cell ordering. Dimensionality reduction and trajectory construction were executed via the reduceDimension function, followed by visualization of gene expression pattern on pseudo-time axis using the plot_genes_in_pseudotime function.

### Definition of EMT macro states

Using the mclust R package 40, we modeled predicted pseudo-time scores as a mixture of three Gaussian distributions, with curve intersection points serving as data-driven boundaries to classify EMT progression into three distinct states: EMT-*early*, EMT-*stable*, and EMT-*late*.

### Inference of metastatic potential and survival/functional dependency

We employed a K-nearest neighbors (KNN) approach to project cell line transcriptomic data onto single-cell differentiation trajectories. Batch effect correction was performed by merging cell line and single cell count matrices followed by the ComBat normalization from sva R package. Using the get.knn function from FNN R package 41, each cell line was mapped to its k-nearest single-cell neighbors along the trajectory. Through systematic evaluation of k-values, we determined k≥50 reliably produced three distinct peaks in the pseudo-time density distribution. Cell line pseudo-time estimates were derived from median values of the 50 nearest single cells, with EMT phase assignment based on established pseudo-time boundaries, thereby facilitating identification of phase-specific metastatic competencies and survival-associated genes.

We extracted the gene knock down profiles generated by shRNA interference across 4,371 target genes and 20 cell lines from LINCs. However, due to the absence of ESCC cell lines, we employed a connectivity method to generate simulated perturbation profiles. We first generated the disease signature using tumor and adjacent normal samples from our bulk RNA-seq cohorts (XJ and TCGA). Subsequently, we computed connectivity scores between disease signature and each perturbation signature using the connectivity map (CMAP) method. Finally, for perturbation signatures across multiple treatment cell lines, concentrations, and times, the signature closest to the median connectivity score was selected to assign a unique signature for each knock down gene.

### Spatial transcriptomic preprocessing

We first loaded the feature-barcode matrix and corresponding high-resolution tissue image using the Load10X_Spatial function. Data normalization was performed via SCTransform with retention of the top 2,000 variable features, followed by PCA dimensionality reduction for downstream analyses. For quality control, the localOutliers function from the SpotSweeper R package 42 was used to identify and remove low quality spots: (i) low library size, (ii) few detected unique features, or (iii) high mitochondrial gene content percentage.

### Inference of copy number alterations

To delineate the tumor regions within our spatial transcriptomics dataset, we used the STARCH Python package 43 designed to infer copy number alterations (CNAs). STARCH identifies tumor clones (setting K = 2 clones) and non-tumor spots. It confirms the identification of normal spots by clustering the first principal component into two clusters using K-means. Changing the value of K alters the number of identified tumor clones, but the number of cells labeled as tumor cells remains the same. We then annotated tissue regions in conjunction with manual annotations from the pathologist (Z.N. and Q.Z.).

### Spot deconvolution

We performed cellular deconvolution of spatial transcriptomics dataset using the spacexr R package 44, which requires both spatial transcriptomic data (including gene expression counts and spatial coordinates) and reference single-cell RNA sequencing data with cell type annotations as input. We ran spacexr in doublet mode to predict one or two predominant cell types for each spot while simultaneously generating a weight matrix representing the proportions of all possible cell types. We first conducted cell subtype deconvolution across all spots, then performing EMT-state-specific deconvolution focused on spots containing mesenchymal-like epithelial cells.

### Spatial colocalization analysis

Using a KNN approach, we first identified neighboring spots surrounding each interested spots, then expanded concentric circular zones (radius levels 1-5) outward from the center to define spatially graduated neighborhoods. Comparative analysis of gene/pathway expressions across these zonal partitions revealed distance-dependent patterns: increased expression indicated spatial co-localization with the interested spots, while decreased expression suggested spatial exclusion.

### Development and validation of the deep learning model

To build sample labels, we categorized 175 specimens into high, medium, and low *CACNA1C* expression groups through Gaussian mixture modeling. The corresponding H&E-stained whole-slide images were randomly divided into training and test sets while maintaining a 4:1 ratio, with reproducibility ensured by setting the random seed to 42. Model evaluation employed five-fold cross-validation, where performance metrics were averaged across all test folds.

For digital pathology image management, we established a standardized preprocessing workflow beginning with background correction using combined Gaussian filtering and OTSU, followed by tissue segmentation into non-overlapping 256×256 patches at 40×magnification. Image normalization was subsequently performed using Reinhard method with Z-score standardization of RGB channels to ensure intensity consistency.

Our analytical framework incorporated four attention-based multiple instance learning (AMIL) architectures 45 (Attention_mil, Clam_sb, Clam_mb, TransMIL), with feature extraction initialized using CTransPath pretrained weights 46. The models were trained for 32 epochs using cross-entropy loss function and Adam optimizer with an initial learning rate of 1×10⁻⁴, with the best-performing model from cross-validation selected for subsequent analysis.

To investigate the clinical relevance of model attention patterns, we extracted patch-level features from the optimal AMIL model's average pooling layer and conducted unsupervised clustering analysis using the Seurat package. Computational efficiency was maintained by subsampling 200 representative patches per specimen for dimensional reduction and cluster visualization.

## Results

### Pathological image analysis reveals an EMT-driven prognostic subtype

**Figure 1:**
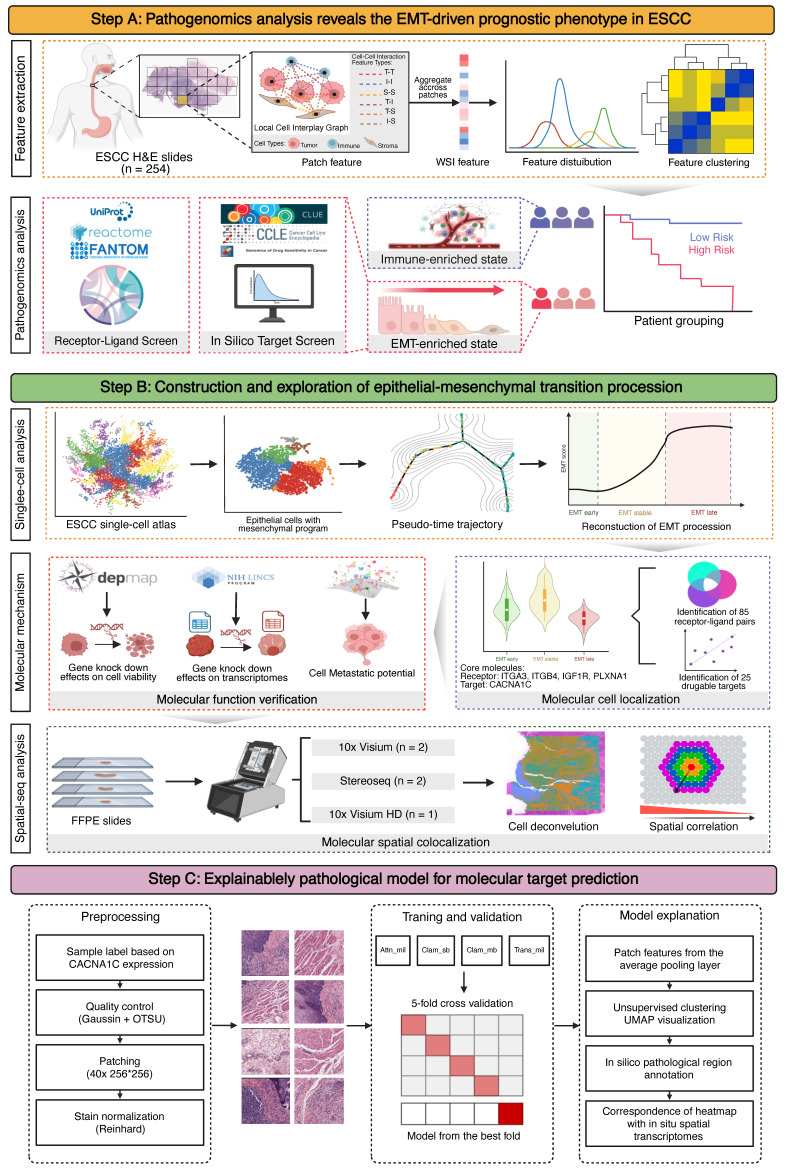
** Schematic workflow of this study.** (A) The hematoxylin & eosin (H&E)-stained whole-section images (WSIs) of esophageal squamous cell carcinoma (ESCC) used in this study are part of routine clinical practice. We used a cell segmentation model to extract pathohistological features from each WSI. Unsupervised clustering revealed pathological subtypes with prognostic value. Bulk-seq data elucidated the molecular dynamics and tumor microenvironment (TME) landscapes of each subtype, allowing us to screen functional ligand-receptor pairs and therapeutic targets in combination with publicly available data. EMT, epithelial-to-mesenchymal transition. (B) Reconstruction and validation of ESCC-specific EMT trajectories. Using single-cell pseudotime analysis, we delineated three macro-EMT states. We combined cell line experimental data to identify core molecules that play central roles in EMT progression and validated their spatial colocalization via spatial transcriptome data. (C) Building a deep learning pathology model for predicting the expression and distribution of molecular targets. We elucidated its interpretability by unsupervised clustering of and matching the predictive attention heatmaps with *in situ* spatial transcriptome data.

**Figure 2:**
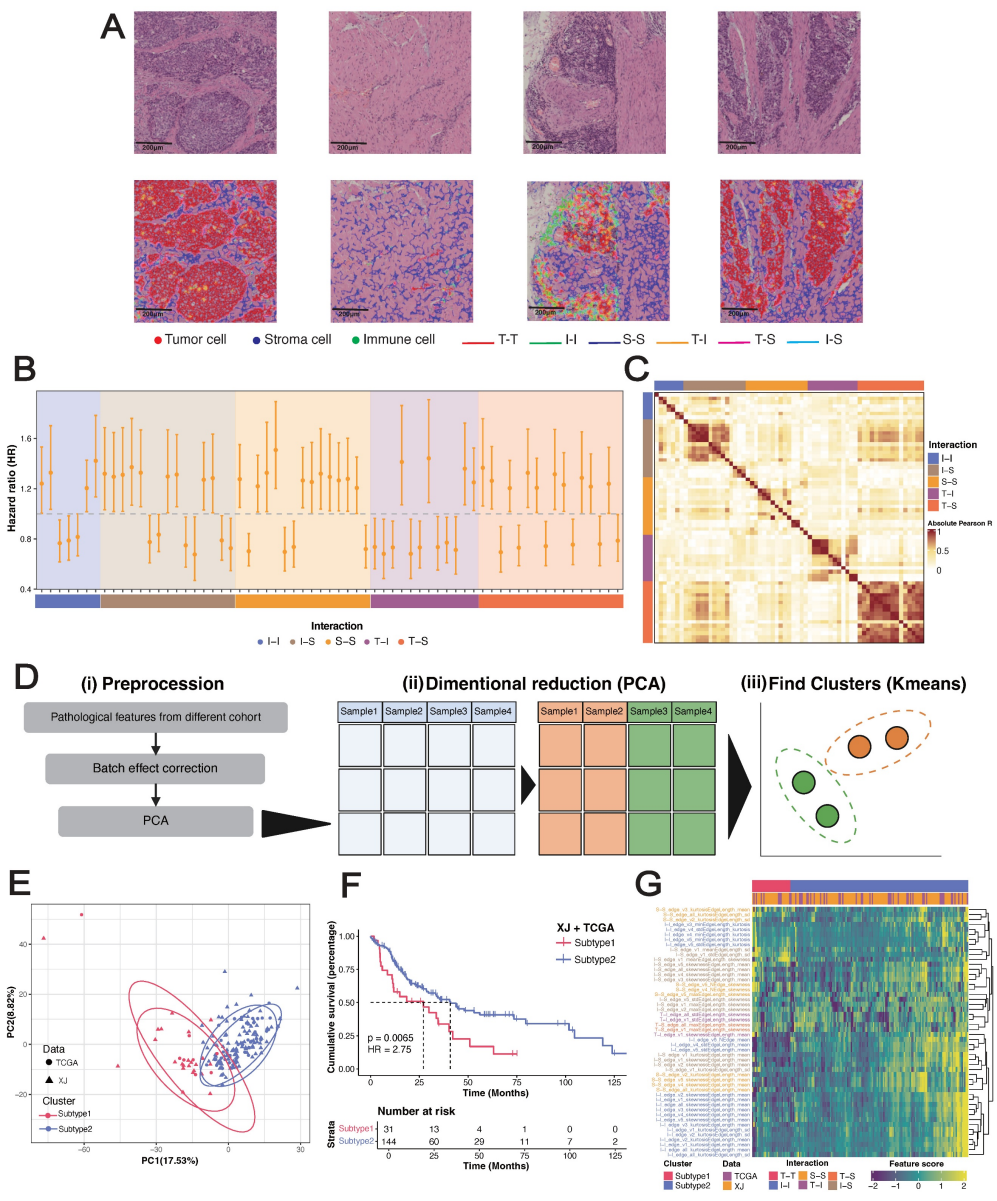
** Pathological analysis reveals prognostic phenotypes in esophageal squamous cell carcinoma (ESCC).** (A) Local nuclear graph visualization of the tumor cells, immune cells, stroma cells and edges among them. (B) Significant univariable associations of pathological features with overall survival in the XJ cohort. (C) Heatmap of correlations between significant prognostic features in the XJ cohort. (D) Workflow of unsupervised clustering. (E) Principal component visualization of the unsupervised clustering result from the merged dataset. (F) Kaplan-Meier analysis of the pathological subtypes. (G) Top 50 contributing pathological features in the principal component analysis (PCA).

Clinically relevant cell types, distributions, and interactions in pathological images were characterized by deep learning-based cell segmentation and spatial mapping. Following strict inclusion criteria (see Methods), we retrospectively obtained 254 H&E-stained whole slide images (WSIs) of primary surgical resection specimens from 254 ESCC patients across three cohorts (XJ = 125, GDPH = 78, TCGA = 50) along with their clinical information (Figure 1A). The WSIs were segmented into 4096×4096 patches at 40x magnification. For each patch, cell segmentation was performed to identify tumor cells (T), immune cells (I), and other stromal cells (S), and a local cell interaction map was constructed using the minimum spanning tree (Figure 2A). Edges longer than 50 pixels were discarded, and up to five edges were constructed per cell (denoted from v1 to v5). We quantified the distribution of edge length using six statistical methods (minimal, maximum, mean, variance, skewness, and kurtosis), generating a multiparameter phenotypic descriptor for each patch. These patch-level descriptors were further aggregated into WSI-level features using four statistical measures (mean, variance, skewness, and kurtosis) for subsequent sample-level analysis.

To investigate the clinical relevance of pathological features, we assessed their associations with overall survival (OS) in the XJ cohort via a univariate Cox regression model (Figure 2B, Table S2). Five types of cell interaction features (I-I, I-S, S-S, T-I, and T-S) were proven to be independent prognostic factors for ESCC. By combining feature interaction and aggregation types, we performed enrichment analysis and noted that immune cell features (I-I_sd, I-S_kurtosis, and I-S_sd) correlated with favorable survival, whereas stromal cell features (S-S_sd, T-S_kurtosis) correlated with poorer outcomes (Figure S1A-B) and had the greatest variability across samples (S-S_skewness, T-S_skewness) (Figure S1C, Table S3). Correlation analysis revealed strong associations between the same feature type and weak associations across different types (Figure 2C), and the T-S features demonstrated the strongest independence. Previous studies have shown that the interplay between cancer-associated fibroblasts (CAFs) and epithelial cells can promote malignant transformation and contribute to the formation of an immunosuppressive TME in ESCC 47, 48. The 65 significant prognostic features were then used to construct a LASSO-Cox regression proportional hazards model to obtain a continuous risk score. This model significantly stratified patients into two risk groups: the XJ cohort (HR = 2.75, 95% CI = [1.70, 4.45], log-rank *p* < 0.0001), the TCGA cohort (HR = 2.55, 95% CI = [1.07, 6.10], log-rank *p* = 0.022), and the GDPH cohort (HR = 2.21, 95% CI = [1.05, 4.65], log-rank *p* = 0.039) (Figure S1D-F). To analyze the biological basis of stratification, we calculated the correlation between the risk scores and the gene set variance analysis (GSVA) scores of the hallmark collection. The top 5 pathways were the coagulation cascade, KRAS upregulation, apoptosis, angiogenesis, and epithelial-mesenchymal transition (Figure S1G), which play important roles in the development of ESCC 49-53.

**Figure 3:**
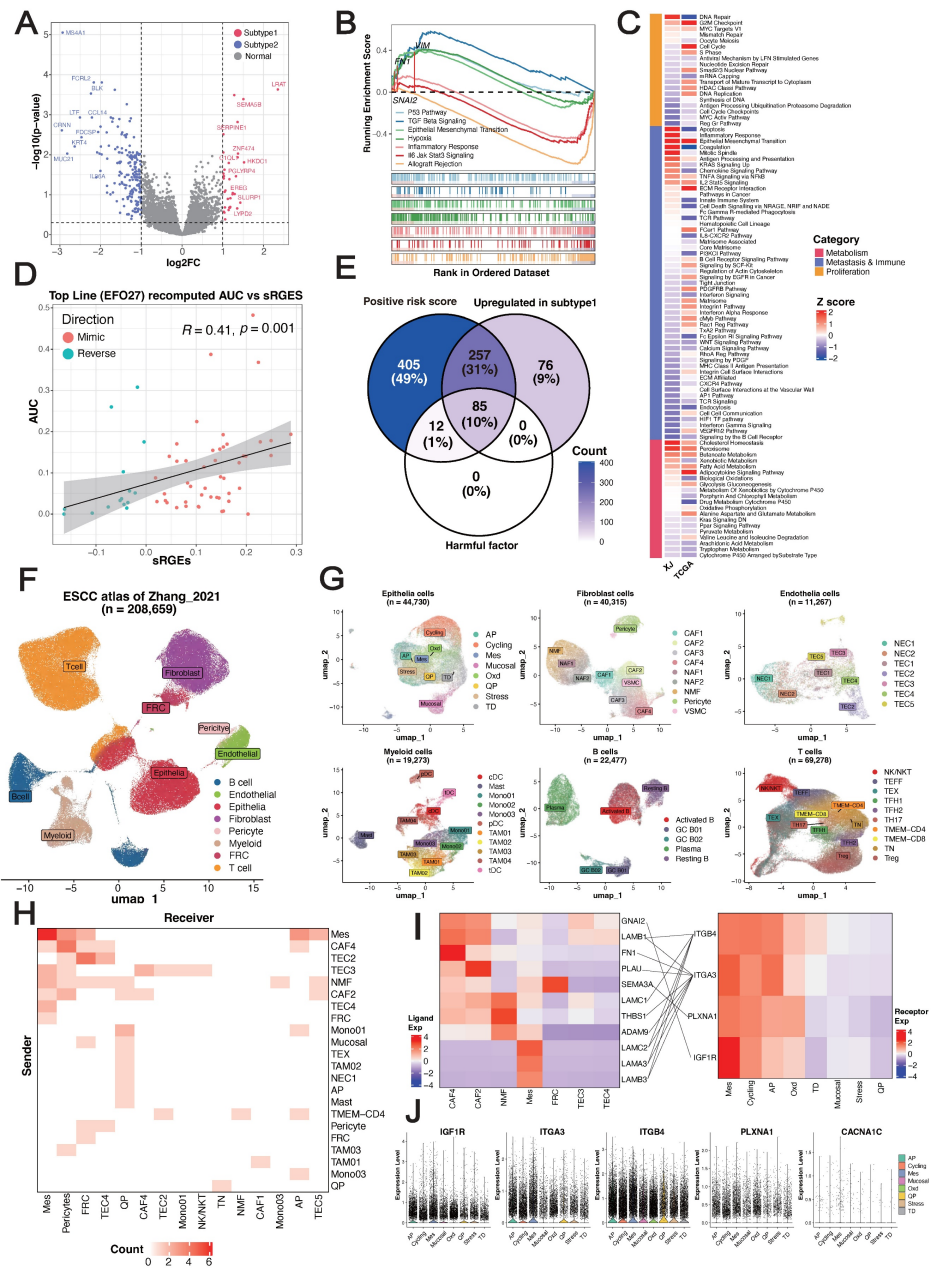
** Tumor microenvironment (TME) and therapeutic characteristics in ESCC prognostic risk stratification.** (A) Volcano plot of differentially expressed genes between pathological subtypes, highlighting the top 20 upregulated and downregulated genes. (B) GSEA of hallmark gene sets. (C) Spearman correlation between the drug response AUC and sRGES in the high-risk subtype representative cell line EFO27. (D) Core receptor-ligand pairs. (E) Heatmap of correlation z score between the predicted pathway scores and GSVA scores. (F-G) UMAP visualization of major cell types and subtypes identified in the Zhang_2021 dataset. (H) Heatmap depicting the cellular subtype localization of core receptor-ligand pairs. (I) Expression heatmap of MES-specific receptors and their corresponding ligands. (J) Expression of MES-specific targets across epithelial cell subtypes.

To further explore the associations of pathological features with clinical prognosis and biological molecules, we merged the XJ and TCGA cohorts (n = 175) with the corresponding bulk-seq profiles. After unsupervised clustering (see Methods), we identified two pathological subtypes with significant overall survival differences (HR = 2.66, 95% CI = [1.75, 4.044], log-rank *p* = 0.0065) (Figure 2D-E). Mirroring our findings from the univariate Cox regression, subtype 1 exhibited elevated stromal cell features and short survival times, whereas subtype 2 presented increased immune cell features and prolonged survival (Figure 2F-G). Differential expression analysis from bulk RNA-seq revealed 754 significantly overexpressed genes in subtype 1 and 1,035 in subtype 2 (|Log2FC| ≥ 1, *p* ≤ 0.05) (Figure 3A). Overrepresentation analysis (ORA) of Gene Ontology terms revealed that cell adhesion, collagen formation, epithelial cell proliferation, and differentiation were specifically upregulated in subtype 1, whereas leukocyte immunity, T-cell differentiation and regulation were upregulated in subtype 2 (Figure S2A). Moreover, gene set enrichment analysis (GSEA) of hallmark samples revealed that subtype 1 was enriched in the EMT and hypoxia pathways in addition to the common *P53* and *TGF-β* pathways, and subtype 2 was enriched in the immune response pathway (Figure 3B). Deconvolution analysis also revealed that the fractions of keratinocytes and immunosuppressive M2 macrophages were increased in subtype 1, whereas those of B cells and T cells were increased in subtype 2 (Figure S2E-F). Whole-exome sequencing (WES) analysis revealed 5 significant somatic mutations (Fisher's exact test *p* < 0.05) (Figure S2C), including *TTN* (*p* = 0.033, 95% CI = [1.00, 23.22]), *PKHD1L1* (*p* = 0.020, 95% CI = [0.04, 0.94]), *EHBP1* (*p* = 0.015, 95% CI = [0.001, 0.81]), LRRC56 (*p* = 0.015, 95% CI = [0.06, 0.81]), and *ZNF429* (*p* = 0.0043, 95% CI = [0, 0.47]). However, the tumor mutation burden (TMB) and fraction of genome altered (FGA) metrics were not significantly different between the subtypes (Figure S2D).

We hypothesized that EMT primarily contributes to the stratification of pathological subtypes. To demonstrate this, we utilized a regularized generalized linear model to learn the associations of pathological features with three types of molecular pathways summarized manually by Jiang* et al.* 54. This analysis revealed that the G2M checkpoint pathway in the proliferation type and the EMT pathway in the migration/immune type were more consistent between cohorts than were pathways in the metabolism type (Figure 3C) (Table S4). Overall, these results indicated that epithelial cells exhibiting high proliferation and differentiation potential, an immunosuppressive TME and enrichment of EMT signatures were characteristic of subtype 1, whereas subtype 2 was related to an immune-activated phenotype and favorable clinical outcomes, and EMT primarily drove the prognostic stratification of pathological subtypes.

### Vulnerability and dependency across pathological subtypes

For the EMT-enriched & high-risk subtype 1, we next sought to identify potential therapeutic agents and targets using a computational approach. The disease signature was defined as a list of differentially expressed genes (DEGs) between tumor and peritumoral samples of subtype 1. By combining drug perturbation transcriptome profiles of cancer cell lines 31, we screened drugs that can inhibit upregulated genes, stimulate downregulated genes, and eventually reverse the gene expression pattern of the disease signature. We acquired a ranked list consisting of 12,443 small-molecule compounds (Table S5). The VEGF-receptor-2-kinase-inhibitor-IV hit was the top-ranked compound with a known mechanism of action 55, 56. In addition, we used the transcriptome profiles to select a cancer cell line most related to subtype 1 and then leveraged the published drug sensitivity data of the representative cell line for *in silico* validation (see Methods). The summarized reverse gene expression scores (sRGES) were significantly positively correlated with the drug efficiency AUCs (Table S6), which increased the confidence of the ranked drug list (Figure 3D). Therefore, we performed enrichment analysis to identify targets whose corresponding drugs were significantly enriched at the top of the prediction. In total, 25 genes were identified as potential targets (NES > 0, *p* ≤ 0.05) (Table S7). Among these genes, the high expression of *CACNA1C,* which encodes an alpha-1 subunit of a voltage-dependent calcium channel, has been proven to be associated with poor differentiation of ESCC 57. Moreover, high expression of its corresponding long-chain noncoding RNA *CACNA1C-AS2* inhibits the proliferation, migration, and invasion of esophageal cancer 58. These findings highlight *CACNA1C* as a potential therapeutic target in ESCC subtype 1.

Considering that the pathological features were derived from a cell-cell interplay map, we next investigated ligand-receptor interactions at the molecular level. On the basis of the manually curated ligand-receptor interaction database 59, we identified 824 pairs in our dataset, with 381 upregulated in subtype 1 and 441 upregulated in subtype 2 (Table S8). Univariate Cox regression revealed a set of 109 pairs significantly associated with OS, with 97 classified as unfavorable prognostic factors and 12 as favorable prognostic factors. Opposite trends for the hazard ratios (negative vs. positive values) were observed for epidermal growth factor receptors (*EGFRs*) and fibroblast growth factor receptors (*FGFRs*), indicating their antagonistic functions (Figure S2G). A summary of the pair-associated pathways revealed that the upregulated pairs in subtype 1 were involved mainly in collagen formation and planar cell polarity, which are also independent unfavorable factors (Figure 2H-I). This analysis enabled us to identify a set of 85 pairs (defined as core pairs, including 42 ligands and 32 receptors) that were positively correlated with the LASSO-Cox risk score, upregulated in subtype 1, and unfavorable factors in terms of prognosis (Figure 3E).

The bulk-seq data includes data on transcriptional programs from a variety of cell types, which could introduce noise signals to our analysis. Therefore, we next validated candidate therapeutic targets and functional ligand-receptor pairs at single-cell resolution (Figure 1B). We incorporated an ESCC single-cell atlas from the Zhang_2021 dataset 60, including 208,659 cells from 64 donors with a total of 128 samples (64 tumors and 64 peritumoral samples). This atlas is represented by 8 major cell types and 51 cell subtypes (Figure 3F-G) (Figure S2J). At the major cell type level, the expression of core pairs was universally upregulated in epithelial cells (Table S8), highlighting their close interactions (Figure S2K). When the signals were refined to the cell subtype level, both the incoming and outgoing signals were dominated by mesenchyme-like epithelial cells (MESs) (Figure 3H) (Figure S2L). Among the core pairs pertaining to epithelial cells (Figure 3I), *LAMA3/LAMB3/LAMC2-ITGA3/ITGB4* were significantly enriched in MESs, indicating their autocrine regulation. In addition, we detected high-frequency interactions between CAFs and MESs: CAF4 and CAF2 could interact with *ITGA3^+^*MES through *FN1* and *PLAU*, respectively, and *LAMB1^+^*CAF4 could interact with both *ITGA3^+^*MES and *ITGB4^+^*MES. Although many genes were not detected, we confirmed that 14 therapeutic candidates were specifically upregulated in epithelial cells (Table S9). Strikingly, *CACNA1C* was the only significantly highly expressed gene in the MESs (logFC = 2.087, *p* < 0.001) (Figure 3J). Overall, we identified *CACNA1C* as a promising therapeutic target for the EMT-enriched subtype 1 subtype and revealed widespread autocrine interactions and paracrine crosstalk of MESs.

### Reconstruction and exploration of EMT trajectories

**Figure 4:**
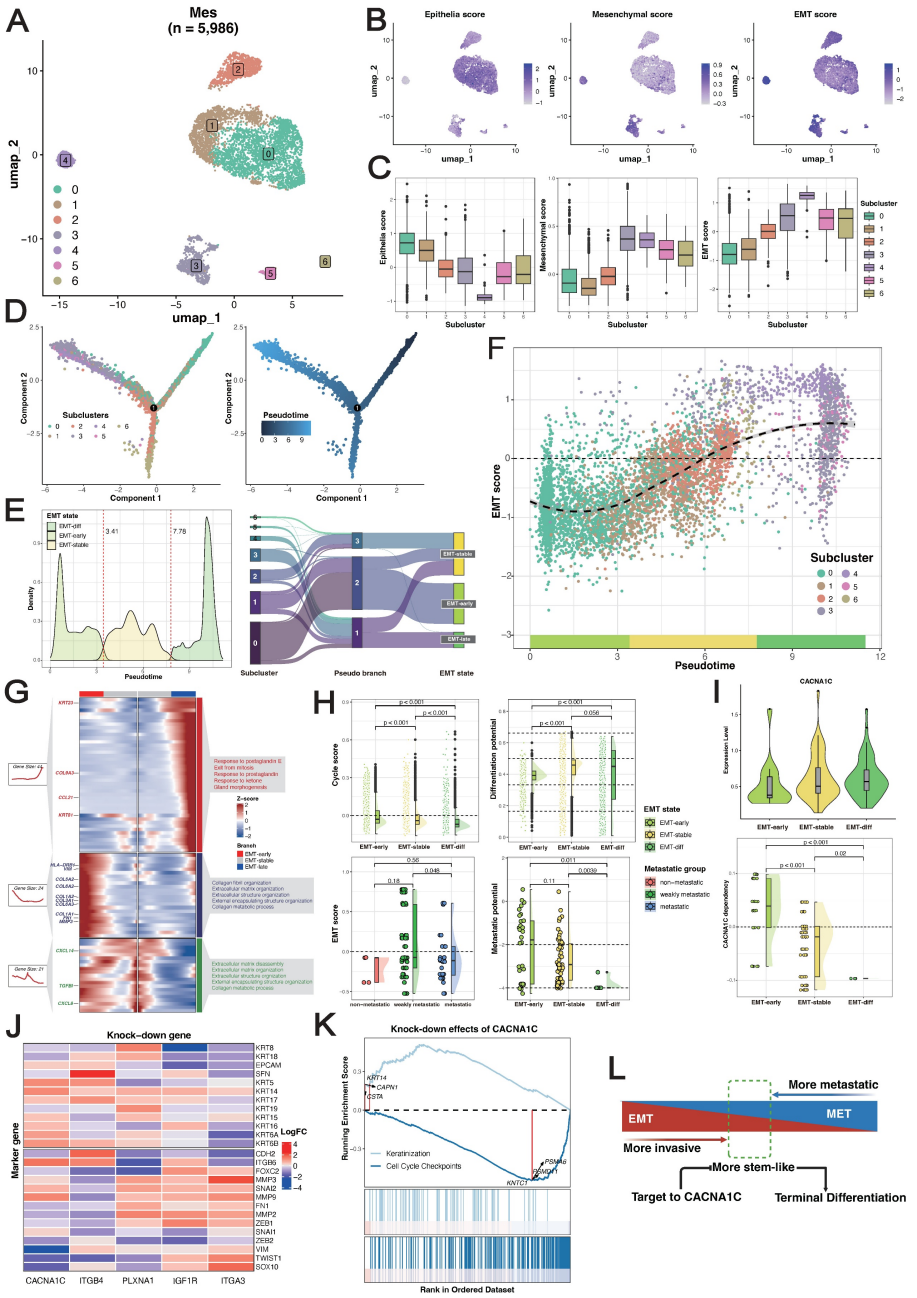
** Reconstruction and exploration of epithelial-to-mesenchymal transition (EMT) trajectories in ESCC.** (A) UMAP visualization of MES subclusters identified from the Zhang_2021 dataset. (B-C) Expression patterns of phenotype marker genes across MES subclusters. (D) Differentiation trajectories of MESs. (E) Gaussian mixture modeling defining three EMT states (left), and relationships between MES subclusters, pseudotime states, and EMT states (right). (F) Landscape of EMT trajectories. (G) Heatmap of branch-dependent genes during state transitions. (H) Comparison of proliferative capacity (upper-left) and stemness (upper-right) across EMT states, EMT scores across metastatic groups in ESCC cell lines (bottom-left), and metastatic potential across EMT states (bottom-right) via the Wilcoxon signed-rank test. (I) Expression localization (upper) and survival dependency (bottom) of *CACNA1C* during EMT. A lower dependency score means that a gene is more likely to be dependent on a given cell line. (J) Effects of gene knockdown on the expression of keratins and collagens. (K) GSEA of genes differentially expressed after *CACNA1C* knockdown using the Reactome gene sets, highlighting the pathways associated with the most significantly up- and downregulated genes. (L) Schematic diagram of *CACNA1C*-targeting mechanisms.

We focused on 5,986 MESs from the Zhang_2021 dataset for further analysis. Unsupervised clustering resulted in seven clusters (Figure 4A). Both the epithelial and mesenchymal programs exhibited cluster-specific enrichment (Figure 4B), resulting in a gradient distribution of EMT scores across clusters (Figure 4C). We inferred the development of MESs by computing a diffusion map and ordering them along a pseudotime axis. Using cluster 0 (with the highest epithelial program) as the origin, trajectory inference predicted two main branches: branch 1 developed through cluster 2 to cluster 1, and branch 2 moved through cluster 3 instead, ending in cluster 4 and cluster 5 (Figure 4D). These trajectories demonstrated that EMT did not follow monotonic linear progression but rather exhibited a stable intermediate state.

To characterize the dominant states governing the continuum of transcriptional activity, we segmented the trajectories on the basis of pseudotime values using a Gaussian mixture model and identified three macro states (Figure 4E): the early state (EMT-*early*), intermediate/residency state (EMT-*stable*), and late state (EMT-*late*). These states were robust to varying pseudotime values, with EMT scores progressively increasing across the three states (Figure 4F) (Figure S3A). Branch-dependent gene analysis revealed regulators governing state transitions (Figure 4G). As expected, keratins (*KRT23/81*) and collagens (*COL1A1/2, COL3A1, COL5A/2,* and* COL6A3*) predominantly shaped epithelial and mesenchymal phenotypes during early and late states, respectively, and *TGFBI* served as the phenotypic stability factor (PSF) for maintaining the EMT-*stable* state. We noted that chemokines (*CCL21, CXCL8/14*) and human leukocyte antigens (*HLA-DRB1*) were highly expressed across the three states, indicating immune system involvement in EMT. Notably, cells in the EMT-*early* state exhibited significantly greater proliferative ability, and cells in the EMT-*stable* state exhibited significantly greater stemness. In terms of their differentiation potential, most MESs maintained lineage-restricted limited differentiation potential (Figure 4H). Recent studies have demonstrated that cells with hybrid E/M phenotypes can either be permanently “locked” in one state or dynamically switch states, which is called epithelial-mesenchymal plasticity (EMP) 61, 62. These results suggested that the ESCC-specific EMT trajectories not only recapitulated classical marker genes but also captured widespread phenotypic plasticity and crosstalk with the immune system, supporting the development of a reliable, generalizable EMT model.

To investigate how EMT states influence metastatic potential, after removing nonbiological batch effects between *in vitro* cell lines and *in vivo* cell models (Figure S3B-C), we projected the corrected metastatic-annotated ESCC cell line profiles onto EMT trajectories (see Methods). Since only epithelial cells were included in the following analysis, confounding effects from mesenchymal cells were precluded. Our analysis robustly captured three EMT macrostates in the cell lines (Figure S3D), with a strong positive correlation between pseudotime values and EMT scores (Figure S3E). As expected, the nonmetastatic group presented relatively lower EMT scores, but the weakly metastatic group presented significantly higher EMT scores than did the metastatic group did (Figure 4H), and we observed a strong negative correlation between EMT scores and metastatic potential across all ESCC cell lines (Figure S3F). This pattern was corroborated by single-cell data, which revealed a decrease in metastatic potential with increasing EMT (Figure 4H) (Figure S3G). The reversal of EMT, a process called mesenchymal-epithelial transition (MET), has been proven to promote metastatic outgrowth at distant sites 63, 64. These findings underscore the importance of homeostasis between epithelial and mesenchymal programs and reveal that cells in the EMT-*early* state, which exhibit a predominant epithelial program, have enhanced metastatic potential.

### *ITGA3* and* ITGB4* exhibit functional antagonism in early EMT

We identified distinct expression patterns of MES-specific receptors associated with different EMT states: *IGF1R* and *PLXNA1* were preferentially expressed during the EMT-*stable* state, whereas *ITGA3* and *ITGB4* were enriched in the EMT-*early* state (Figure S3H). Intriguingly, *ITGA3* depletion concurrently suppressed epithelial programs and activated mesenchymal programs, whereas *ITGB4* depletion induced the opposite effects (Figure 4J). This opposing effect was further evidenced by strongly negatively correlated transcriptome profiles upon perturbation (Figure S3I), indicating fundamental differences in the downstream regulatory consequences. In contrast, *IGF1R* and *PLXNA1* knockout did not significantly affect transcriptome profiles (Figure S4J). Collectively, these findings establish an antagonistic relationship between *ITGA3* and *ITGB4* in the EMT-*early* state, where *ITGB4* promotes EMT and *ITGA3* drives MET. The dynamic balance of these processes determines the differentiation fate of cells in the EMT-*early* state (Figure 4L).

### Inhibition of *CACN1C* reprograms the malignant phenotype

The phenotypic plasticity of MESs and the dynamic nature of epithelial and mesenchymal programs during metastasis highlight a possible weakness of cell transdifferentiation. As *CACN1C* was identified as a promising therapeutic target for the poorly prognostic subtype, we next investigated its function in the EMT trajectory. *CACNA1C^+^*MESs demonstrated a lower expression level in cells in the EMT-*early* state than in those in the EMT-*stable*/*late* states (Figure 4I). Consistently, projection of corrected dependency-annotated ESCC cell line onto EMT trajectories revealed that cells in the EMT-*stable*/*late* states showed significantly greater survival dependency than those in the EMT-*early* state (Figure 4I). We postulated that the elevated expression of *CACN1C* reflected enrichment of cells in the EMT-*stable/late* state, potentially explaining its correlation with the poor differentiation of ESCC. To delineate its mechanism, we further analyzed the gene knockdown transcriptome profiles of the ESCC cell lines. We found that *CACNA1C* depletion concurrently upregulated epithelial programs, downregulated mesenchymal programs (Figure 4J), and significantly suppressed the cell cycle pathway (Figure 4K). Therefore, we hypothesized that targeting *CACN1C* simultaneously inhibits both the mesenchymal program and the proliferative program in cells in the EMT-*stable/late* state and initiates their transdifferentiation into normal epithelium-like cells (Figure 4L).

**Figure 5:**
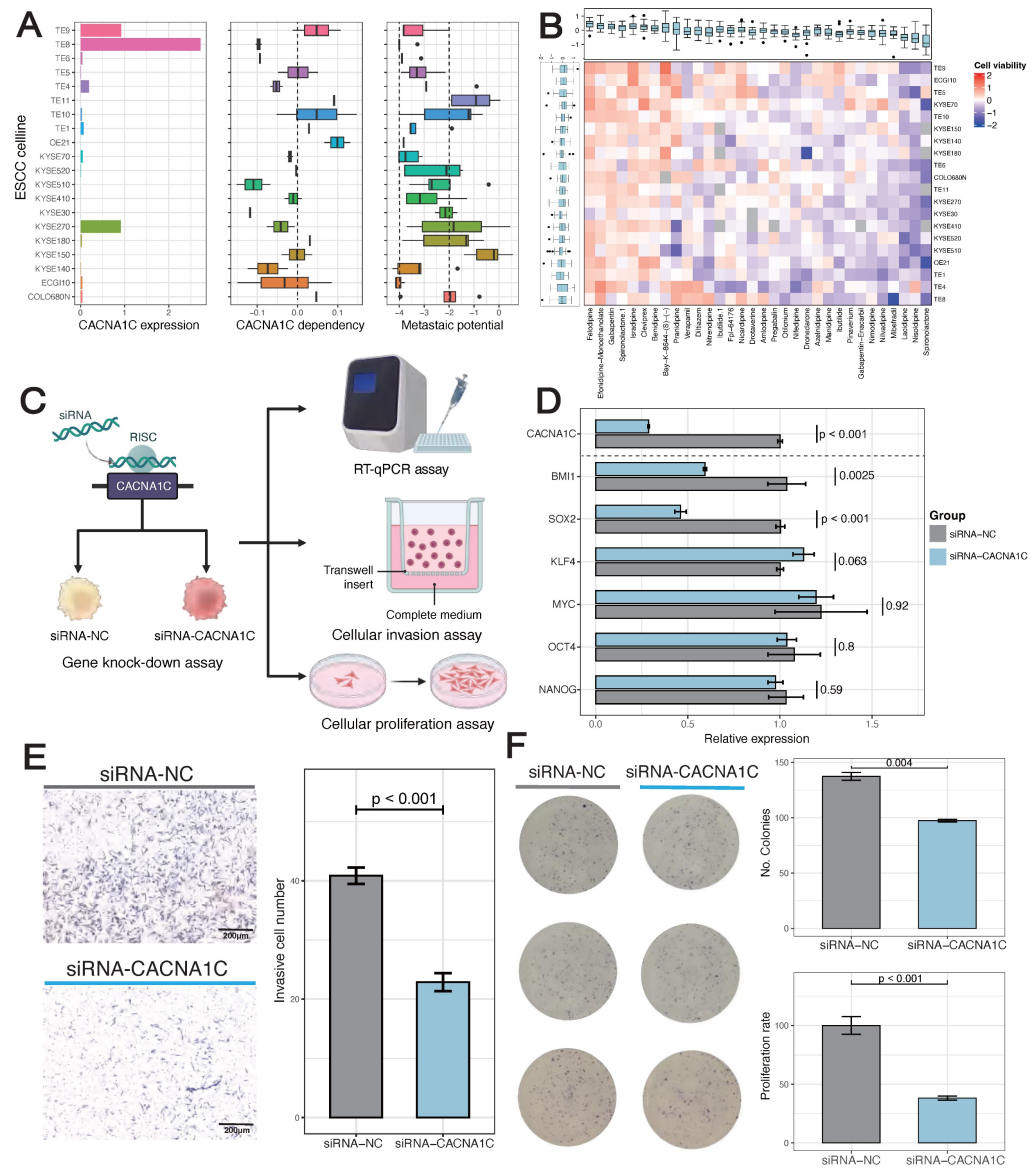
** Inhibition of CACN1C represses stemness, invasion and proliferation of TE-8 human ESCC cell line.** (A) Basic characteristics of ESCC cell line. (B) ESCC cell line viability after disturbance of *CACNA1C* targeted compounds. (C) Scheme of cell line functional experiment. (D) The relative expression of *CACNA1C* and stemness markers in TE-8 cell line expressing siRNAs targeting *CACNA1C* (siRNA-*CACNA1C*) or control siRNA (siRNA-NC). Quantitative PCR with reverse transcription (RT-qPCR) data are normalized to *GAPDH* mRNA expression. n = 6 replicates per group. Statistical analysis was performed using two-sided Student's t-test. (E) Left, Crystal violet staining imaging of invasion assay. Right, Quantification showing the knock-down effect on TE-8 invasion. (F) Left, Crystal violet staining imaging of colony formation assay. Right, Quantification showing the knock-down effect on TE-8 clone formation and proliferation.

Screening of human ESCC cell line from DepMap illustrated the highest basic *CACNA1C* expression and dependency in TE-8, which had weakly metastatic potential (Figure 5A). In addition, TE-8 showed sensitivity to more than half of *CACNA1C* targeted compounds (Cell viability < 0) (Figure 5B). To validate the impact of *CACNA1C* on the malignant phenotype, we performed *in vitro* gene knock-down and functional assays using TE-8 (Figure 5C). The mRNA expression of cancer stemness related genes, including *BMI1*, *SOX2*, *KLF4*, *MYC*, *OCT4*, and *NANOG* were analyzed in paired cell line models with or without knock-down. RT-qPCR showed that *BMI1* and *SOX2* were significantly decrease, and *KLF4* was lightly increased compared with controls (Figure 5D). The activation of *BMI1* and *SOX2* has been reported to be drivers of ESCC 65. *KLF4* is enriched in normal esophageal epithelium, and it has been shown that its expression is lost in ESCC and associated with poor prognosis 66, thus the upregulated of *KLF4* might indicate the tendency of recovery of cell phenotype from malignant to normal. In contrast, the expression of other stemness genes, including *MYC*, *OCT4*, and *NANOG*, remained largely unchanged, which indicated a selective disruption of a specific regulatory module of cancer stemness. Additionally, the chamber invasion assay (Figure 5E) and the colony formation/proliferation assay (Figure 5F) showed that the *CACNA1C* enhanced the invasion and proliferation process of TE-8. These findings collectively highlight the essential role of inhibiting *CACNA1C* in reprogramming ESCC malignant phenotype.

### Spatial colocalization of EMT niches

**Figure 6:**
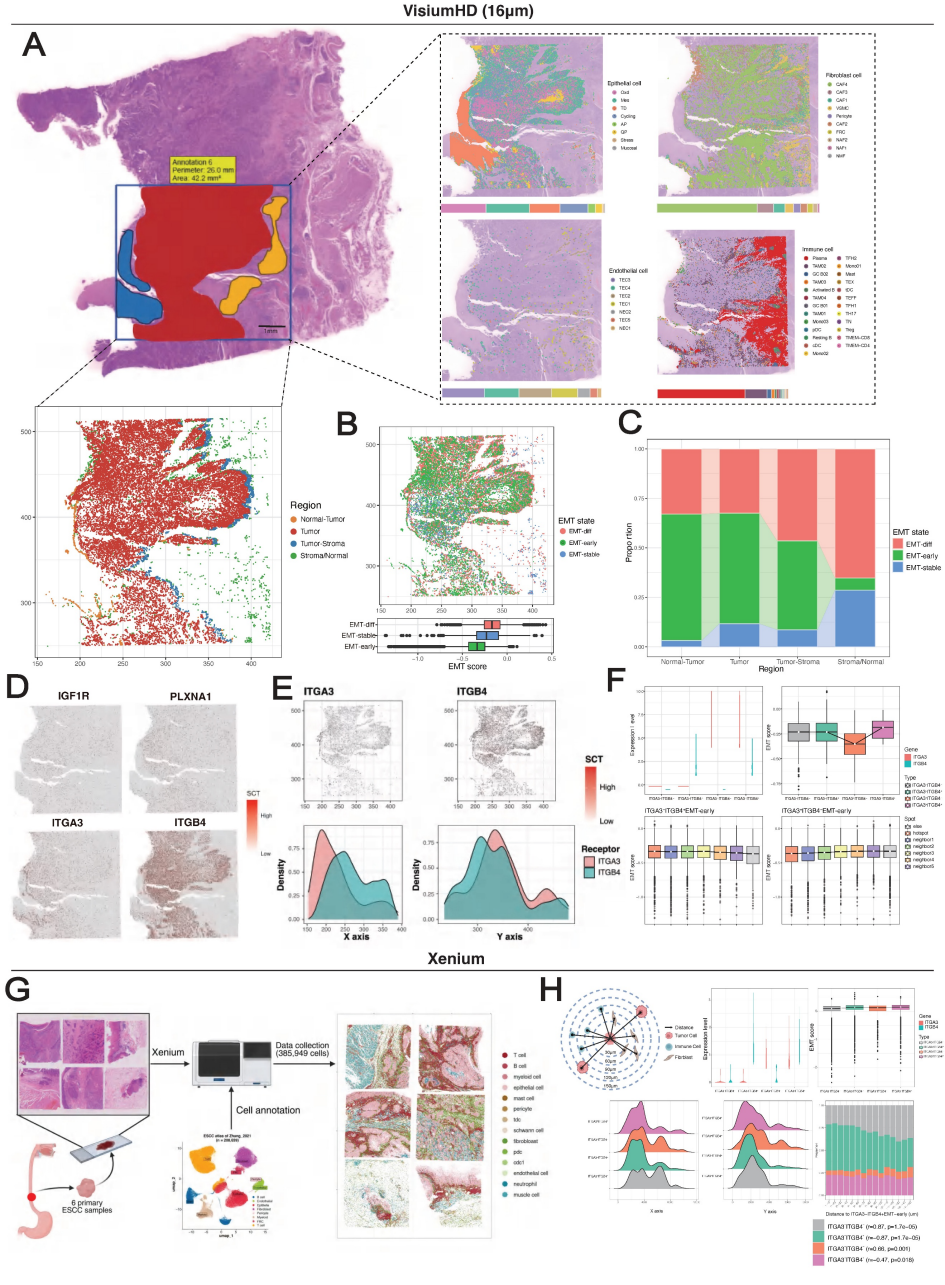
** Spatial cell colocalization along the EMT trajectories.** (A) Spatial mapping of a VisiumHD sample (E0) with 16-μm bins colored by cell type predicted by deconvolution via the single-cell reference dataset (right) and MES-containing spots colored according to tissue region (bottom). (B) Spatial mapping of EMT states. (C) Stacked histogram with the proportions of EMT states across tissue regions. (D) Spatial visualization of MES-specific receptors. (E) Spatial visualization of *ITGA3* and *ITGB4* in MES-containing spots. (F) Spatial correlation between *ITGA3/ITGB4*-specific EMT-*early* spots and regional EMT activity. (G) Schematic of workflows for Xenium experiment. (H) Top, Diagram showing the quantification of spatial proximity of cells, and violin and bar plot showing EMT-*early* subtype classification. Bottom, Kernel density plots displaying the EMT-*early* distribution along X and Y axis, and stacked bar plots showing the proportion of EMT-*early*. *P* values calculated by Mann-Kendall trend test.

Given the extensive interactions among MESs, we hypothesized that their spatial colocalization might establishe EMT niches. We employed high-resolution spatial transcriptomic technologies including Visium HD for discovery (tumor E0: 152,517 bins at 16 μm resolution) and Stereo-seq for verification (tumors E1 and E2: 130,927 and 106,236 bins at 20 bin resolution, respectively). Following a uniform quality control pipeline (Figure S4A) (Figure S5A), we performed spot deconvolution based on single-cell data, which generated a spatial map consistent with the tissue morphology (Figure 6A). Copy number variation (CNV) inference delineated the tumor region, tumor-normal epithelium interface, and normal/stromal regions. With reference to the pathologist-annotated tissue masks, we further defined the tumor-stroma interface as a 3-spot-wide zone adjacent to the tumor boundary.

Oxidative phosphorylation-characterized epithelial cells (OXDs) and MESs were the predominant cell types in the spatial map, and they exhibited distinct zonation patterns. OXDs were localized in the tumor nest, whereas MESs surrounded them in the periphery (Figure 6A). The transcriptomic similarity between MESs and OXDs was corroborated by single-cell UMAP projection (Figure 3I). The application of EMT trajectories to spots containing MESs revealed a reliable spatial map of EMT states (Figure 6B). Intriguingly, we noted the spatial overlap of EMT-*stable* cells and OXDs. Reactive oxygen species (ROS) accumulate during EMT initiation and cancer progression 67, and this spatial association suggests that OXDs may represent an alternative origin distinct from that of EMT-*early* cells. A quantitative comparison of the cell proportions in tissue regions demonstrated the enrichment of cells in the EMT-*early* state at tumor-normal interfaces, while cells in the EMT-*late* state predominated at tumor-stroma interfaces, emphasizing the stroma-oriented invasion of cells in the EMT-*late* state (Figure 6C).

Spatial mapping of MES-specific receptors showed their predominant localization within the tumor region (Figure 6D). Specifically, *ITGA3* exhibited preferential expression toward the normal epithelium, whereas *ITGB4* presented tumor- and stroma-oriented expression (Figure 6E). Spots containing EMT-*early* state cells were classified into four groups based on *ITGA3* and *ITGB4* expression (Figure S4B). As predicted, the *ITGA3^+^ITGB4^-^* spots had the lowest EMT scores, whereas the *ITGA3^-^ITGB4^+^* spots showed significantly higher scores (Figure 6F). Despite the limited spots detected (n = 407), *ITGA3^+^ITGB4^+^* spots presented the highest EMT scores, which was recapitulated by the single-cell data (Figure S4C). These findings collectively suggest a cooperative effect in which *ITGA3* and *ITGB4* are coexpressed in the EMT-*early* state.

Using a neighborhood enrichment approach (see Methods), we evaluated the environment around *ITGA3^+^ITGB4^-^* and *ITGA3^-^ITGB4^+^* EMT-*early* spots (Figure S4E-F). EMT scores decreased progressively with distance from the *ITGA3^-^ITGB4^+^* spots, but showed the opposite trend for the *ITGA3^+^ITGB4^-^* spots (Figure 6F). Using Stereo-seq data, we consistently observed spatial exclusion of *ITGA3* and *ITGB4* (Figure S5D-H), their potential cooperative effects, and distance-dependent EMT distributions (Figure S5I-J). In addition, EMT-*early* specific ligands (*LAMA3*, *LAMB3*, *LAMC2*) showed decreasing expression with distance from both *ITGA3^+^ITGB4^-^* and *ITGA3^-^ITGB4^+^* spots (Figure S4F-G). In contrast, expression of the CAF4-specific ligand *FN1* increased with distance from *ITGA3^+^ITGB4^-^* spots (Figure S4H).

We further employed 10x Xenium technology for in-situ spatial profiling 5,000 mRNAs across 385,959 cells from 6 primary ESCC samples (Figure 6G). After obtaining 14 major cell types using marker genes, we employed label transfer approach to acquire subtypes of epithelial and fibroblast cells using single-cell data as reference (Table S6). Subsequently, we distinguished EMT-early subtypes by the expression levels of *ITGA3* and *ITGB4*, and calculated spatial distances between cells at single-cell resolution. In brief, we not only verified the spatial exclusion between ITGA*3*^+^*ITGB4*^-^ and *ITGA3*^-^*ITGB4*^+^ EMT-early (Figure 6H), but also the spatial co-localization between *ITGA3*^+^*ITGB4*^-^ EMT-early with CAF4 (Figure S6). Taken together, these results collectively suggest that cells in the EMT-*early* state colocalize with self-derived or CAF4-secreted ligands to establish EMT niches.

### Prediction of *CACN1C* expression and distribution using pathological H&E images

Deep learning has been successfully applied in various cancer types for extracting clinically relevant features from routine histopathological slides 68-70. Building on the above findings, we subsequently investigated whether deep learning could accurately predict molecular target expression and whether computational predictions would reveal meaningful clinical and molecular associations.

**Figure 7:**
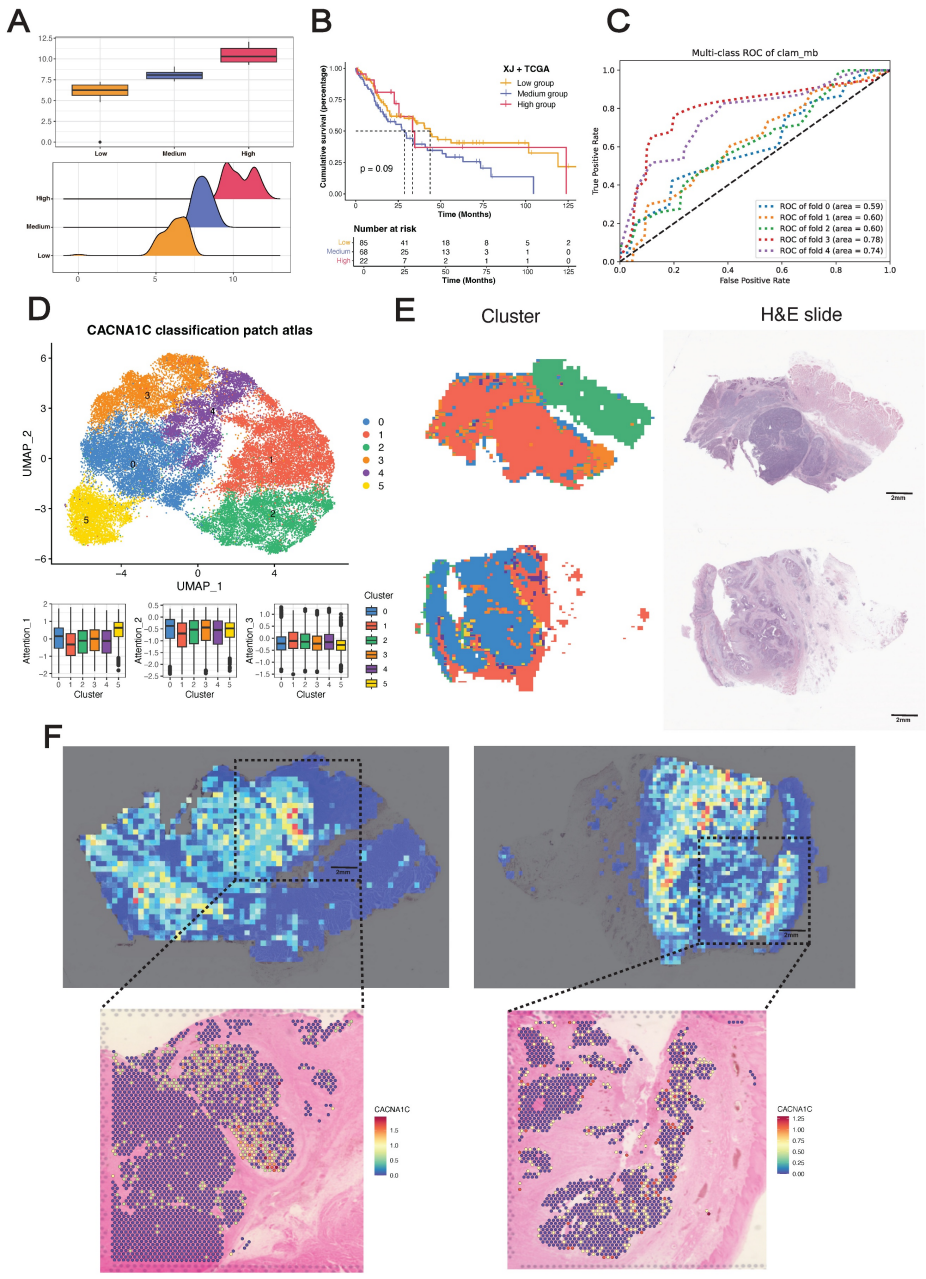
** Development and validation of the pathological deep learning model.** (A) Sample classification on the basis of *CACNA1C* expression. (B) Kaplan-Meier analysis of the sample classification. (C) Receiver operating characteristic (ROC) curves showing the performance of the Clam_mb model in fivefold cross-validation. (D) UMAP visualization of patch-level clusters derived from deep learning features, with box plots quantifying each cluster's contribution to attention scores. (E) Spatial distribution mapping of computational patch clusters across whole-slide images. (F) Spatial concordance between the model attention score and *in situ CACNA1C* expression in tumor tissues.

We initially discretized the *CACNA1C* expression values into three groups (high/medium/low) as sample labels (Figure 7A), with significant differences in OS between the medium- and low-expression groups, whereas the high-expression group presented a fluctuating Kaplan-Meier curve (Figure 7B), highlighting the biological complexity of ESCC prognosis. We subsequently trained several attention-based multiple instance learning (AMIL) models for three-class classification (Figure 7C) (Figure S7), among which Clam_mb demonstrated superior predictive performance in our dataset, achieving a mean fivefold cross-validated area under the receiver operating characteristic curve (AUROC) of 0.668, outperforming other AMIL models (Clam_sb = 0.658, Attention_mil = 0.648, Trans_mil = 0.628).

To decipher the clinical implications of model predictions, we extracted patch-level features from the average pooling layer of the Clam_mb model, reduced feature dimensionality to 50 using PCA, and identified six patch clusters using Louvain clustering method (Figure 7D). These clusters demonstrated differential attention score contributions: cluster 5 showed the highest attribution for low-expression classification, cluster 0 for medium-expression classification, and cluster 1 for high-expression classification. Whole-slide visualization and H&E-correlated annotation revealed distinct histopathological identities: cluster 0 represented stromal tissue, cluster 1 represented the tumor region, cluster 2 represented muscle/normal epithelium, cluster 3 represented the tumor stroma, and clusters 4 and 5 represented invasive tumor margins (Figure 7E). Collectively, these computationally derived clusters presented unique histopathological signatures, suggesting the tissue-specific expression of *CACNA1C* in ESCC.

To gain further insights into the *in situ* relationships between the model predictions and the biological ground truth, we performed Visium sequencing on two ESCC FFPE tissue sections (capturing 4,820 and 4,468 spots from tumors E3 and E4, respectively), with tumor/stroma demarcation through CNV inference. Despite the inherent tissue morphology variations between serial sections, application of the Clam_mb model to adjacent H&E-stained sections revealed strong concordance between attention heatmaps and spatial transcriptomics, where model-identified high-attention regions exhibited corresponding gene-specific upregulation (Figure 7F). Notably, we did not incorporate tissue masks or region-specific cropping during training, and the model selectively focused on high-expression regions in tumor areas while filtering out stromal signals (Figure S7D), demonstrating that our deep learning framework can sensitively detect *in situ CACNA1C* expression patterns in tumor tissue from H&E images.

## Discussion

Understanding the mechanisms by which cell crosstalk drives cancer aggression and metastasis is critical for improving the diagnostic precision and development of targeted therapies for ESCC. Here, we described the development, validation, and explanation of pathological cell-cell interplay features for prognosticating overall survival time using H&E-stained images. We combined a deep learning model for cell segmentation and classification, a minimum spanning tree for topological feature construction and extraction, a Cox regression model and unsupervised clustering to maximize interpretability. We developed and validated our features using three independent cohorts. Our results revealed that image-derived cell interaction features serve as strong predictors of survival outcomes in ESCC patients and are concordant with known molecular pathways. The clinical and biological relevance of these features support their utility and generalizability.

Using combined bulk-seq data, we demonstrated the existence of a high-risk pathological subtype characterized by an EMT-enriched state and emphasized the concomitant immunosuppressive microenvironment. In addition, the ligand-receptor pairs enriched in pathological subtypes demonstrated EMT-promoting effects, with predominant localization between mesenchymal cell-like epithelial cells (MESs) and between MESs and CAFs, indicating both autocrine and paracrine regulation of EMT. Through systematic target screening, we identified *CACNA1C* as a specifically overexpressed target in MESs, which is consistent with its established association with poor ESCC differentiation.

EMT progression has been proven to be a continuous phenotypic process characterized by multiple intermediate states 71. However, to our knowledge, relevant investigations have relied on pancancer cell line models, leaving a critical gap in single-cell characterization using clinical specimens. We reconstructed EMT trajectories using the largest available single-cell atlas of clinical ESCC samples. Gradient shifts between epithelial and mesenchymal programs across MESs demonstrated phenotypic continuity, and pseudotime analysis revealed a plateau state during EMT progression. On the basis of pseudotime score distributions, we defined three macrostates for MESs (EMT-*early*, EMT-*stable*, and EMT-*late*), with progressively increasing EMT scores across states. As anticipated, keratins and collagens mainly shape epithelial and mesenchymal phenotypes in the early and late states, respectively, whereas *TGFBI* functions as a phenotypic stability factor during the stable phase. Notably, chemokines and human leukocyte antigens exhibit stage-dependent expression, indicating that there is crosstalk between EMT and the immune system. Furthermore, cells in the EMT-*early* state displayed the greatest proliferative capacity, cells in the EMT-*stable* state presented the highest differentiation potential, and most MESs maintained lineage-restricted limited differentiation potential, collectively demonstrating remarkable phenotypic plasticity during EMT. The full spectrum of EMT intermediate states remains to be fully characterized, and the mechanism of state switches needs to be better understood. Here, the ESCC-specific EMT trajectory delineates three well-supported macrostates reflecting both intrinsic cancer cell alterations and microenvironment adaptations.

It has been reported that cells with hybrid E/M phenotypes generate progeny cells that are either mesenchymal or epithelial and are more prone to migrate 72. To evaluate the impact of EMT on ESCC metastasis, we projected metastatic ESCC cell lines onto EMT trajectories. This analysis revealed that cells in the EMT-*early* state had the strongest metastatic potential, highlighting the critical balance between the epithelial and mesenchymal programs. While enrichment of the epithelial program is required for ESCC metastasis, we demonstrated that targeting *CACNA1C* simultaneously suppresses both the mesenchymal program and cell cycle progression in EMT-*stable/late* states, driving their transdifferentiation into normal epithelium-like cells. Furthermore, we established that *ITGB4* overexpression promotes EMT, whereas *ITGA3* enhances MET. Importantly, by exclusively analyzing epithelial cells, we eliminated potential confounding effects from mesenchymal cell contamination.

Recent studies have demonstrated that hybrid E/M cells preferentially localize to the tumor invasive front 73-75. Similarly, our spatial transcriptomic data revealed enrichment of EMT-*early* cells at the tumor-normal epithelium interface, whereas EMT-*late* cells were predominant at the tumor-stroma border. Notably, we observed spatial overlap between EMT-*stable* and epithelial cells with oxidative phosphorylation characteristics (OXDs), suggesting that OXDs may serve as alternative EMT-initiating cells distinct from those with *early EMT*. Previous work revealed that *ITGA3* and *ITGB1* are specifically overexpressed in the tumor-specific keratinocytes (TSKs) of cutaneous squamous cell carcinoma, where they function as receptors for ligands expressed by TAMs and MDSCs 76. Our findings not only confirmed the mutually exclusive spatial expression of *ITGA3* and *ITGB4* but also revealed a potential cooperative role in driving EMT progression when they are coexpressed in the EMT-*early* state.

End-to-end deep learning models have yielded impressive results for diagnostic applications such as the detection of cancer and the prediction of the primary origin of metastases 77, 78. Attention-based MIL methods learn from patient-level labels and offer explanations in the form of saliency heatmaps that localize relevant regions. Inspired by Calderaro *et al*. 79, we used spatial transcriptomes as the gold standard for the molecular detection problem. We developed a target prediction model in which computationally derived patch clusters exhibited distinct histopathological signatures, with *CACNA1C*-high expression clusters specifically localized to tumor regions. Spatial validation demonstrated strong concordance between the model predictions and the *in situ* biological ground truth. Notably, without incorporating tissue masks or region-specific cropping during training, the model autonomously attended to tumor-specific high-expression zones, confirming both the tissue-specific expression of *CACNA1C* and the translational potential of this model.

This study has some limitations: (i) As a retrospective analysis of patient cohorts, this study requires future prospective validation to confirm its predictive findings. (ii) Although local cell interaction maps effectively correlate with clinical and biological features, the absence of detailed tissue architecture and refined cellular subtypes may limit analytical depth. This limitation could be addressed through dedicated tissue and cell segmentation models. (iii) ESCC-specific EMT trajectories warrant comprehensive validation in clinical metastatic samples, and the time-course dynamics should be further explored. (iv) Model performance should be improved by several approaches, including expanding training datasets, employing more robust pre-training encoders, obtaining accurate labels via *in situ* hybridization, combining multiple pathological images from the sample patient and multimodal feature engineering.

Overall, the results of this study systematically delineate EMT dynamics in ESCC through integrative analysis of histopathology, bulk-seq, single-cell transcriptomics, and spatial transcriptomics. By analyzing cellular interaction features from pathological images, we first demonstrated that pathological patterns are driven by EMT dynamics. We further validated the continuum of intermediate EMT states, revealed the pivotal role of the EMT*-early* state in metastasis and further identified *CACNA1C* as a therapeutic target for cells in the EMT-*stable/late* state. These findings provide novel insights into the cellular and molecular mechanisms underlying tumor invasion and metastasis. Finally, we developed an end-to-end deep learning model that predicts therapeutic targets from pathology images, enabling clinically translatable risk stratification and personalized therapy for ESCC patients.

## Supplementary Material

Supplementary figures.

Supplementary tables.

## Abbreviations

ESCC: esophageal squamous cell carcinoma
TME: tumor microenvironment
EMT: epithelial-mesenchymal transition
WSI: whole slide image
OS: overall survival
CAFs: cancer-associated fibroblasts
MESs: mesenchyme-like epithelial cells
MET: mesenchymal-epithelial transition
CNV: copy number variation
AMIL: attention-based multiple instance learning
