# Supplementary material for: Pathogenomic analysis reveals clinically relevant epithelial-mesenchymal plasticity in esophageal squamous cell carcinoma
Source: Theranostics. 2026 Jan 14;16(7):3870–91. doi: 10.7150/thno.125381 (PMC12846784; doi:10.7150/thno.125381)
Supplement: Supplementary file 1 — Supplementary figures. [file thnov16p3870s1.pdf]

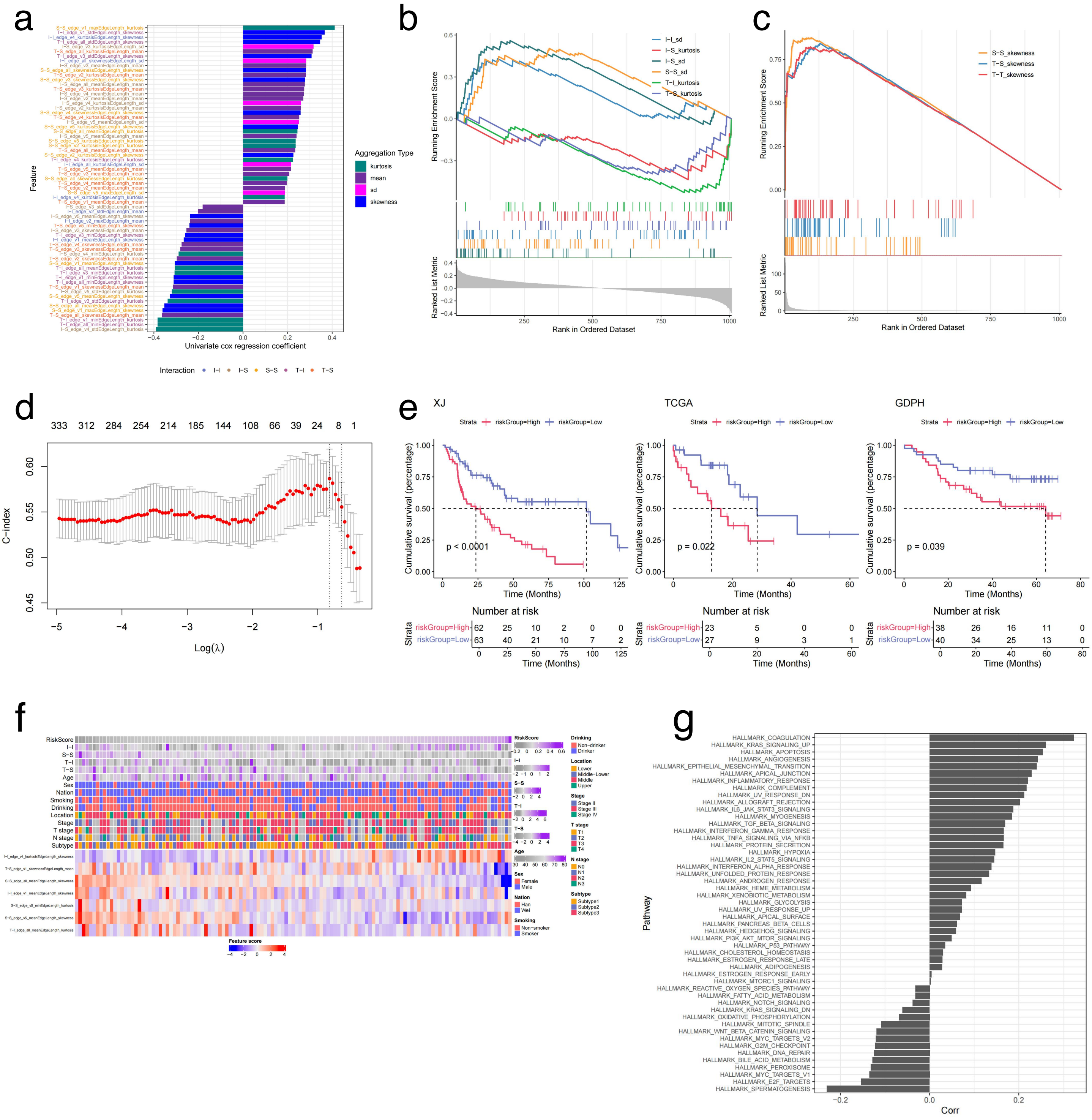
a
Feature
Univariate cox regression coefficient
Aggregation Type
kurtosis
mean
sd
skewness
Interaction
I−I
I−S
S−S
T−I
T−S
b
Running Enrichment Score
I−I_sd
I−S_kurtosis
I−S_sd
S−S_sd
T−I_kurtosis
T−S_kurtosis
Ranked List Metric
Rank in Ordered Dataset
c
Running Enrichment Score
S−S_skewness
T−S_skewness
T−T_skewness
Ranked List Metric
Rank in Ordered Dataset
d
C−index
Log(λ)
333 312 284 254 214 185 144 108 66 39 24 8 1
e
XJ
TCGA
GDPH
Strata
riskGroup=High
riskGroup=Low
Cumulative survival (percentage)
Time (Months)
p < 0.0001
p = 0.022
p = 0.039
Number at risk
f
RiskScore
I−I
S−S
T−I
T−S
Age
Sex
Nation
Smoking
Drinking
Location
Stage
T stage
N stage
Subtype
Feature score
g
Pathway
Corr
HALLMARK_COAGULATION
HALLMARK_KRAS_SIGNALING_UP
HALLMARK_APOPTOSIS
HALLMARK_ANGIOGENESIS
HALLMARK_EPITHELIAL_MESENCHYMAL_TRANSITION
HALLMARK_APICAL_JUNCTION
HALLMARK_INFLAMMATORY_RESPONSE
HALLMARK_COMPLEMENT
HALLMARK_UV_RESPONSE_DN
HALLMARK_ALLOGRAFT_REJECTION
HALLMARK_INTERFERON_GAMMA_RESPONSE
HALLMARK_IL6_JAK_STAT3_SIGNALING
HALLMARK_MYOGENESIS
HALLMARK_TGF_BETA_SIGNALING
HALLMARK_UNFOLDED_PROTEIN_RESPONSE
HALLMARK_TNFA_SIGNALING_VIA_NFKB
HALLMARK_PROTEIN_SECRETION
HALLMARK_HYPOXIA
HALLMARK_IL2_STAT5_SIGNALING
HALLMARK_INTERFERON_ALPHA_RESPONSE
HALLMARK_UNFOLDED_PROTEIN_RESPONSE
HALLMARK_ANDROGEN_RESPONSE
HALLMARK_HEME_METABOLISM
HALLMARK_XENOBIOTIC_METABOLISM
HALLMARK_GLYCOLYSIS
HALLMARK_UV_RESPONSE_UP
HALLMARK_APICAL_SURFACE
HALLMARK_PANCREAS_BETA_CELLS
HALLMARK_HEDGEHOG_SIGNALING
HALLMARK_PI3K_AKT_MTOR_SIGNALING
HALLMARK_P53_PATHWAY
HALLMARK_CHOLESTEROL_HOMEOSTASIS
HALLMARK_ESTROGEN_RESPONSE_LATE
HALLMARK_ADIPOGENESIS
HALLMARK_ESTROGEN_RESPONSE_EARLY
HALLMARK_MTORC1_SIGNALING
HALLMARK_REACTIVE_OXYGEN_SPECIES_PATHWAY
HALLMARK_FATTY_ACID_METABOLISM
HALLMARK_NOTCH_SIGNALING
HALLMARK_KRAS_SIGNALING_DN
HALLMARK_OXIDATIVE_PHOSPHORYLATION
HALLMARK_MITOTIC_SPINDLE
HALLMARK_WNT_BETA_CATENIN_SIGNALING
HALLMARK_MYC_TARGETS_V2
HALLMARK_G2M_CHECKPOINT
HALLMARK_DNA_REPAIR
HALLMARK_BILE_ACID_METABOLISM
HALLMARK_PEROXISOME
HALLMARK_MYC_TARGETS_V1
HALLMARK_E2F_TARGETS
HALLMARK_SPERMATOGENESIS

Figure S1. Prognostic risk stratification based on pathological features.

(a) Bar plot of significant prognostic features (univariate cox p-value < 0.05), color-coded by aggregation method (bars) and feature type (y-axis). (b) Enrichment analysis of feature risk scores. (c) Enrichment analysis of feature variable coefficients. (d) Concordance index (C-index) trajectories during five-fold cross-validation. (e) Kaplan-Meier survival curves demonstrating model performance across three independent cohorts. (f) Heatmap displaying model-used features with aligned clinical annotations. (g) Correlation analysis between model risk scores and Hallmark pathway GSVA values, presented as a ranked bar plot.

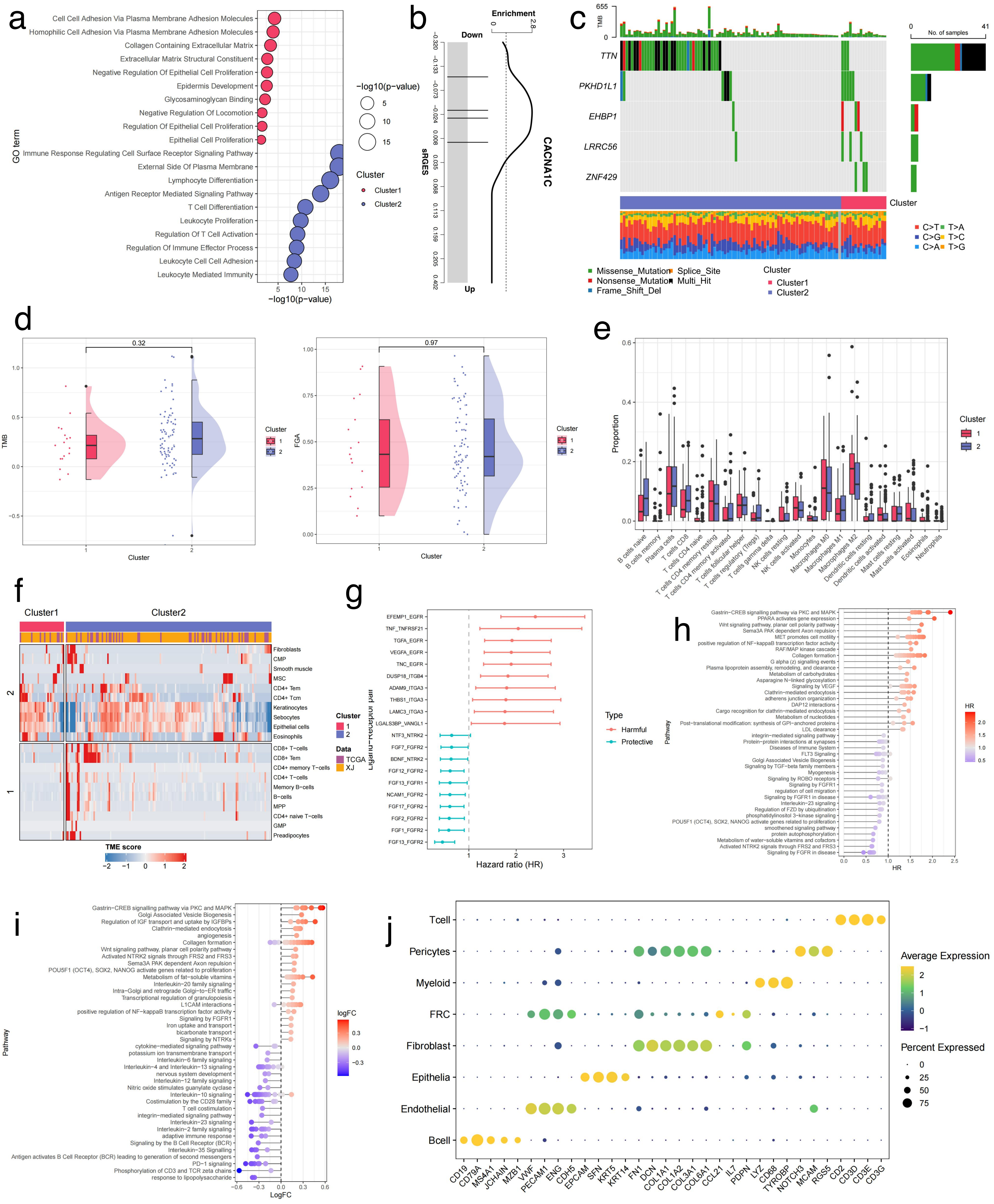

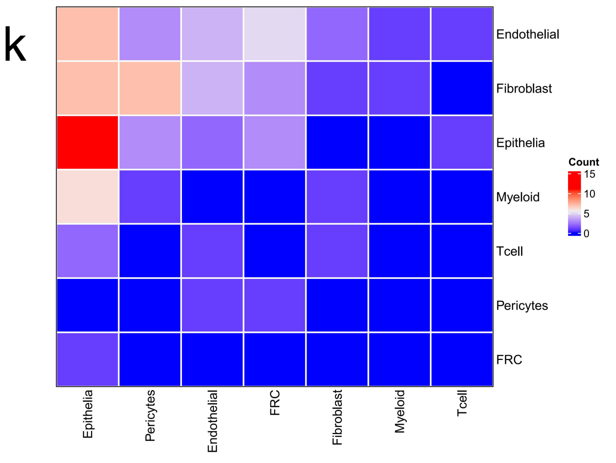

l

Cell	Receptor_count	Ligand_count
Epithelia	34	20
Pericytes	14	2
Endothelial	12	23
FRC	12	1
Fibroblast	5	23
Myeloid	2	8
Tcell	2	4

m

Cell	Receptor_count	Ligand_count
Mes	15	16
Pericytes	13	2
FRC	11	1
QP	10	1
AP	7	1
TEC4	5	2
TEC5	4	NA
CAF4	3	8
TEC2	2	7
NMF	1	8
Mono01	1	3
Mono03	1	1
TN	1	NA
CAF1	1	NA
NK/NKT	1	NA
TEC3	NA	8
CAF2	NA	6
TMEM−CD4	NA	3
Mucosal	NA	2
Mast	NA	1
NEC1	NA	1
TEX	NA	1
Pericyte	NA	1
TAM02	NA	1
TAM01	NA	1
TAM03	NA	1

Figure S2. Molecular dynamics of prognostic risk stratification in esophageal squamous cell carcinoma (ESCC).

(a) ORA using GOBP terms, showing the top 10 up- and down-regulated terms. (b) CACNA1C enriched profile. (c) Significantly mutated genes between pathological subtypes. (d) Tumor mutational burden and fraction of genome altered values across pathological subtypes. (e-f) Cell abundance of pathological subtypes predicated by Cibersort and xCell. (g) Forest plot of significant prognostic receptor-ligand pairs, showing the top 10 protective and hazardous pairs. (h-i) Hazard ratio (HR) and LogFC distribution of receptor-ligand pairs in pathway contexts. (j) Canonical markers for major cell types. (k) Heatmap mapping receptor-ligand pairs to their predominant cellular sources. (l-m) Cellular localization frequency counts for receptor-ligand pairs at major cell type and subtypes resolution.

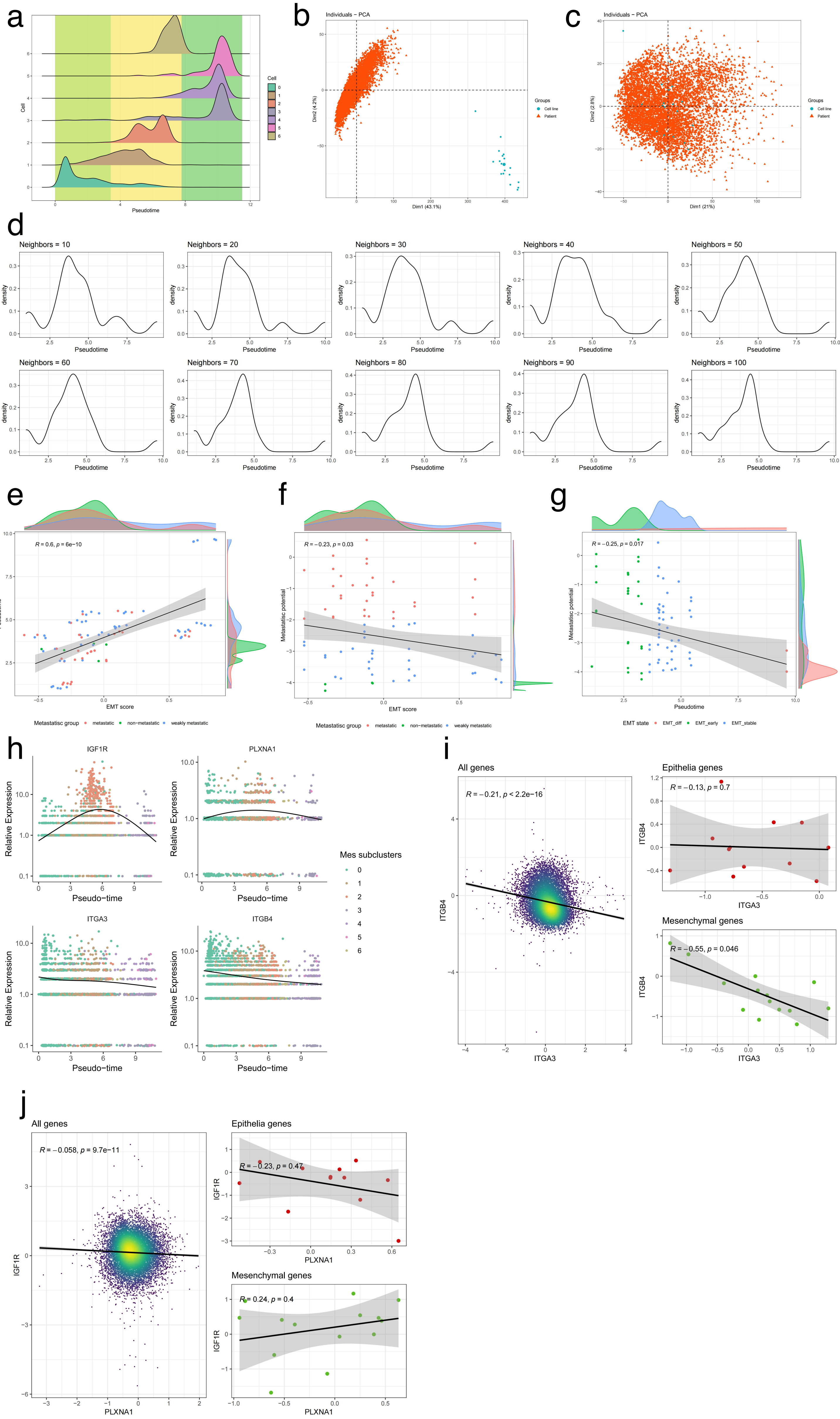
a
Cell
Pseudotime
b
Individuals - PCA
Dim1 (43.1%)
Dim2 (4.2%)
Groups
Cell line
Patient
c
Individuals - PCA
Dim1 (21%)
Dim2 (2.8%)
Groups
Cell line
Patient
d
Neighbors = 10
Neighbors = 20
Neighbors = 30
Neighbors = 40
Neighbors = 50
Neighbors = 60
Neighbors = 70
Neighbors = 80
Neighbors = 90
Neighbors = 100
density
Pseudotime
e
R = 0.6, p = 6e−10
Pseudotime
EMT score
Metastatisc group
metastatic
non−metastatic
weakly metastatic
f
R = −0.23, p = 0.03
Metastatisc potential
EMT score
Metastatisc group
metastatic
non−metastatic
weakly metastatic
g
R = −0.25, p = 0.017
Metastatisc potential
Pseudotime
EMT state
EMT_diff
EMT_early
EMT_stable
h
IGF1R
PLXNA1
ITGA3
ITGB4
Relative Expression
Pseudo-time
Mes subclusters
i
All genes
R = −0.21, p < 2.2e−16
ITGB4
ITGA3
Epithelia genes
R = −0.13, p = 0.7
ITGB4
ITGA3
Mesenchymal genes
R = −0.55, p = 0.046
ITGB4
ITGA3
j
All genes
R = −0.058, p = 9.7e−11
IGF1R
PLXNA1
Epithelia genes
R = −0.23, p = 0.47
IGF1R
PLXNA1
Mesenchymal genes
R = 0.24, p = 0.4
IGF1R
PLXNA1

Figure S3. Validation of epithelial-to-mesenchymal transition (EMT) trajectories. (a) Pseudo-time score distribution across MESs. (b-c) PCA of integrated cell line and single-cell data before and after batch effect correction. (d) Pseudo-time score distribution under varying KNN parameters. (e-g) Correlation analyses between: EMT score and pseudo-time, EMT score and metastatic potential, and pseudo-time and metastatic potential. (h) State-specific expression patterns of MES-specific receptors. (i-j) Knockdown signature correlation between: *ITGA3* and *ITGB4*, *IGF1R* and *PLXNA1*.

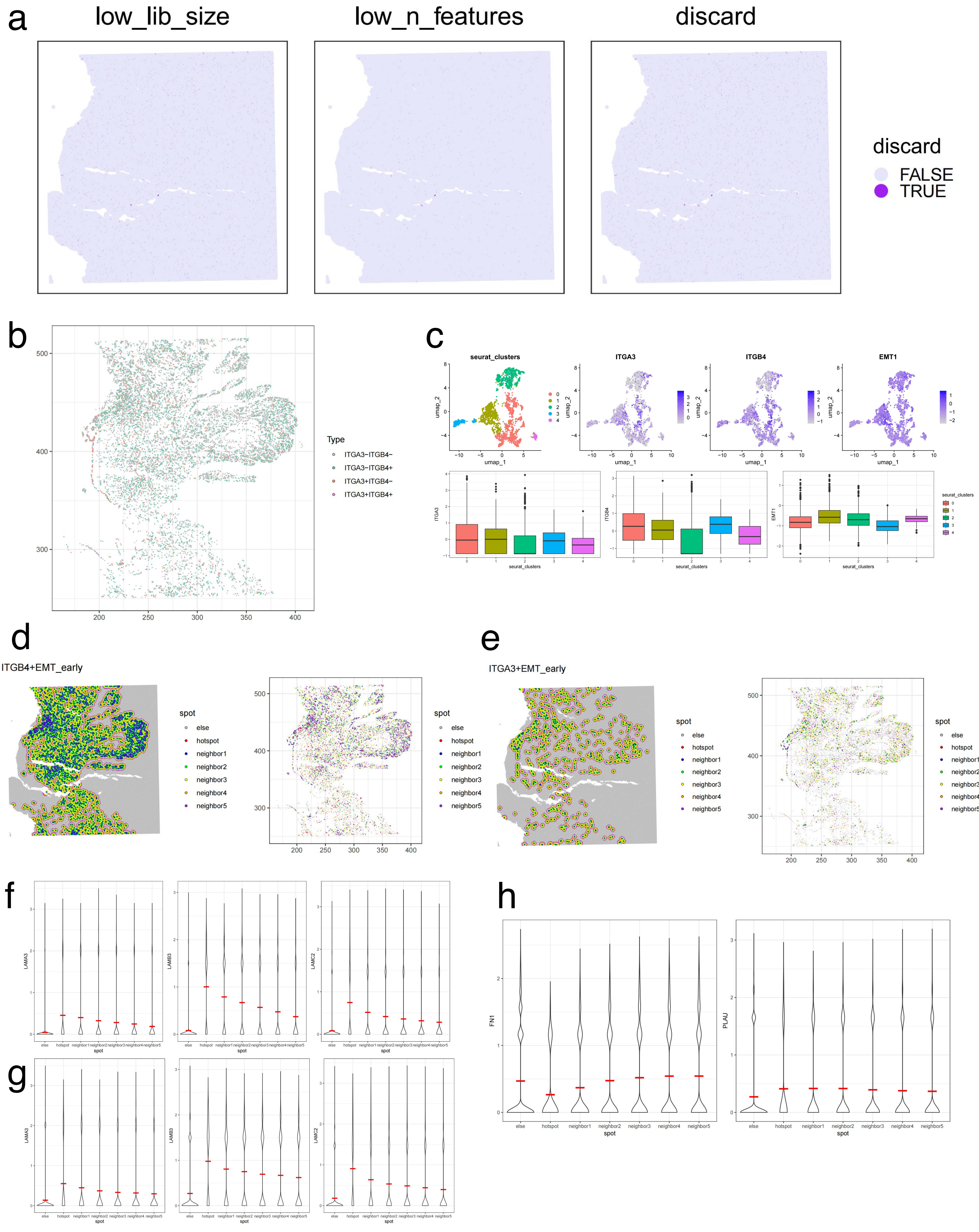

a
low_lib_size
low_n_features
discard
discard
FALSE
TRUE
b
Type
ITGA3−ITGB4−
ITGA3−ITGB4+
ITGA3+ITGB4−
ITGA3+ITGB4+
c
seurat_clusters
ITGA3
ITGB4
EMT1
umap_1
umap_2
d
ITGB4+EMT_early
spot
else
hotspot
neighbor1
neighbor2
neighbor3
neighbor4
neighbor5
e
ITGA3+EMT_early
f
g
h
LAMA3
LAMB3
LAMC2
FN1
PLAU

Figure S4.Quality control and co-localization in Visium HD data.

(a) Quality-control metrics for the Visium HD data. Spots with low library size and low number of detected unique features were discarded. (b) Spatial mapping of EMT-early across the tissue section. (c) UMAP visualization and box plot of EMT-early. (d) $ITGB4^{+}$ EMT-early with their neighbors in all spots and in EMT-early spots. (e) $ITGA3^{+}$ EMT-early with their neighbors in all spots and in EMT-early spots. (f) Co-localization of $ITGB4^{+}$ EMT-early with their autocrine ligand. (g) Co-localization of $ITGA3^{+}$ EMT-early with their autocrine ligand. (h) Co-localization of $ITGA3^{+}$ EMT-early with CAF4-specific ligands.

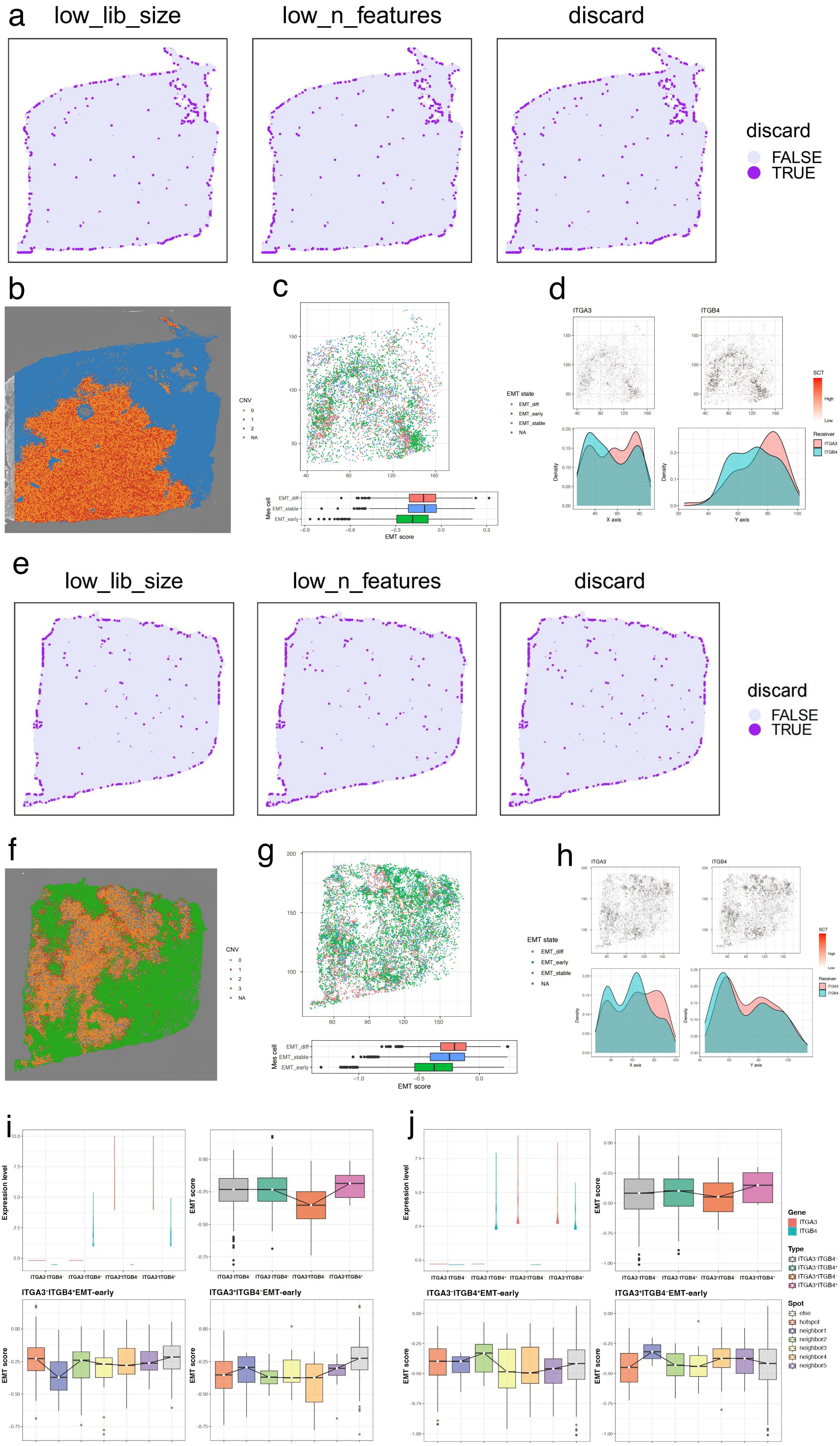

a
low_lib_size
low_n_features
discard
discard
FALSE
TRUE
b
CNV
0
1
2
NA
c
EMT state
EMT_diff
EMT_early
EMT_stable
NA
Mes cell
EMT_diff
EMT_stable
EMT_early
EMT score
d
ITGA3
ITGB4
SCT
High
Low
Receiver
ITGA3
ITGB4
Density
X axis
Y axis
e
low_lib_size
low_n_features
discard
discard
FALSE
TRUE
f
CNV
0
1
2
3
NA
g
EMT state
EMT_diff
EMT_early
EMT_stable
NA
Mes cell
EMT_diff
EMT_stable
EMT_early
EMT score
h
ITGA3
ITGB4
SCT
High
Low
Receiver
ITGA3
ITGB4
Density
X axis
Y axis
i
Expression level
EMT score
ITGA3⁻ITGB4⁻
ITGA3⁻ITGB4⁺
ITGA3⁺ITGB4⁻
ITGA3⁺ITGB4⁺
ITGA3⁻ITGB4⁺EMT-early
ITGA3⁺ITGB4⁻EMT-early
j
Expression level
EMT score
ITGA3⁻ITGB4⁻
ITGA3⁻ITGB4⁺
ITGA3⁺ITGB4⁻
ITGA3⁺ITGB4⁺
ITGA3⁻ITGB4⁺EMT-early
ITGA3⁺ITGB4⁻EMT-early
Gene
ITGA3
ITGB4
Type
ITGA3⁻ITGB4⁻
ITGA3⁻ITGB4⁺
ITGA3⁺ITGB4⁻
ITGA3⁺ITGB4⁺
Spot
else
hotspot
neighbor1
neighbor2
neighbor3
neighbor4
neighbor5

Figure S5. Quality control and co-localization in Stereo-seq data.

(a, e) Quality-control metrics for the Stereo-seq data in E1 and E2 samples. (b, f) CNA inference results. (c, g) Spatial mapping of EMT states. (d, h) Spatial visualization of *ITGA3* and *ITGB4*. (i, j) Spatial correlation between *ITGA3/ITGB4*-specific EMT-*early* spots and regional EMT activity

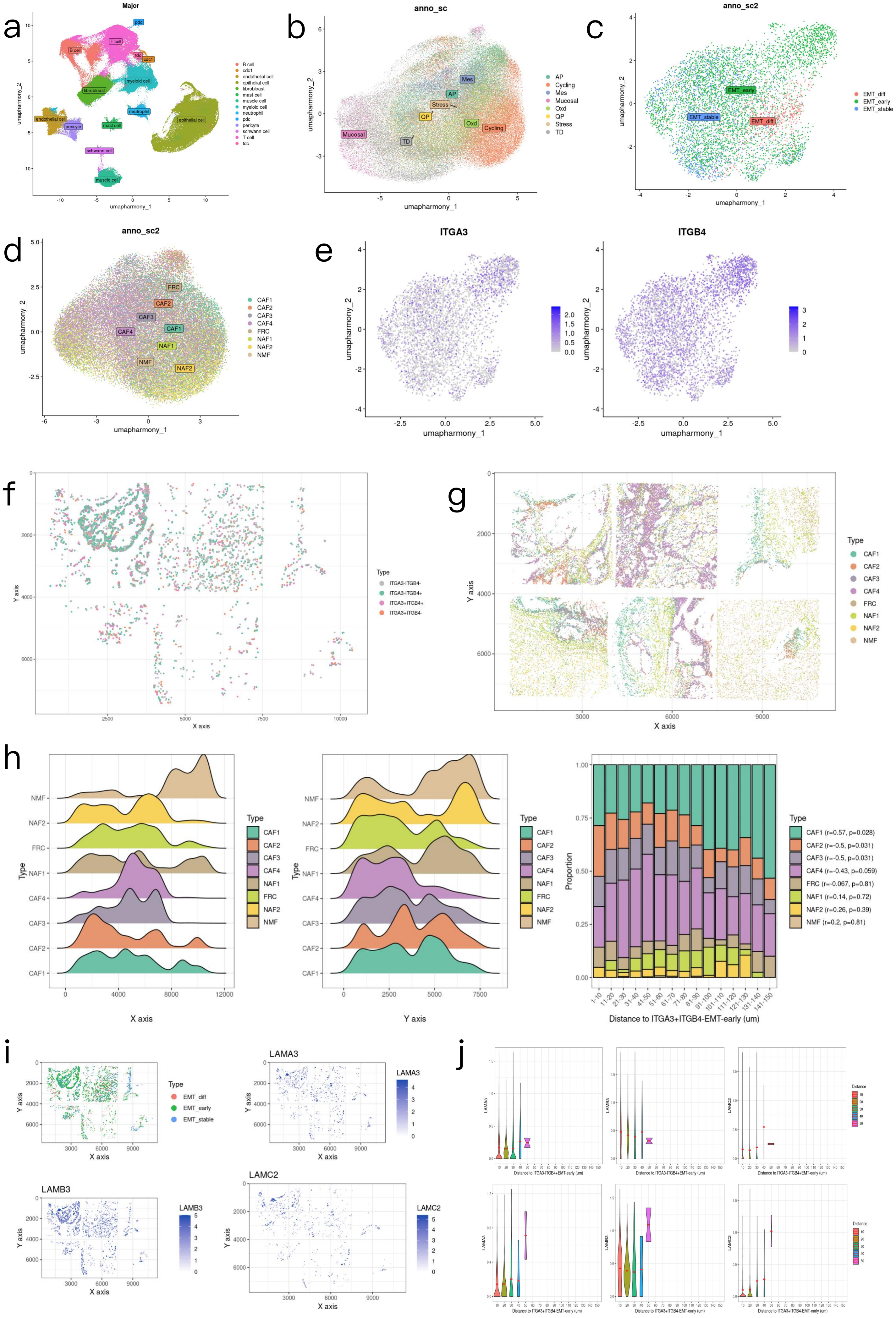

a
Major
B cell
T cell
pdc
tdc
cdc1
myeloid cell
fibroblast
neutrophil
epithelial cell
endothelial cell
pericyte
mast cell
schwann cell
muscle cell
umapharmony_1
umapharmony_2
B cell
cdc1
endothelial cell
epithelial cell
fibroblast
mast cell
muscle cell
myeloid cell
neutrophil
pdc
pericyte
schwann cell
T cell
tdc
b
anno_sc
Mes
AP
Stress
QP
Oxd
Cycling
Mucosal
TD
umapharmony_1
umapharmony_2
AP
Cycling
Mes
Mucosal
Oxd
QP
Stress
TD
c
anno_sc2
EMT_early
EMT_stable
EMT_diff
umapharmony_1
umapharmony_2
EMT_diff
EMT_early
EMT_stable
d
anno_sc2
FRC
CAF2
CAF3
CAF1
CAF4
NAF1
NMF
NAF2
umapharmony_1
umapharmony_2
CAF1
CAF2
CAF3
CAF4
FRC
NAF1
NAF2
NMF
e
ITGA3
ITGB4
umapharmony_1
umapharmony_2
f
Type
ITGA3-ITGB4-
ITGA3-ITGB4+
ITGA3+ITGB4+
ITGA3+ITGB4-
X axis
Y axis
g
Type
CAF1
CAF2
CAF3
CAF4
FRC
NAF1
NAF2
NMF
X axis
Y axis
h
Type
CAF1
CAF2
CAF3
CAF4
NAF1
FRC
NAF2
NMF
X axis
Y axis
Proportion
Distance to ITGA3+ITGB4-EMT-early (um)
CAF1 (r=0.57, p=0.028)
CAF2 (r=-0.5, p=0.031)
CAF3 (r=-0.5, p=0.031)
CAF4 (r=-0.43, p=0.059)
FRC (r=-0.067, p=0.81)
NAF1 (r=0.14, p=0.72)
NAF2 (r=0.26, p=0.39)
NMF (r=0.2, p=0.81)
i
Type
EMT_diff
EMT_early
EMT_stable
LAMA3
LAMB3
LAMC2
X axis
Y axis
j
Distance
Distance to ITGA3-ITGB4+EMT-early (um)
Distance to ITGA3+ITGB4-EMT-early (um)
LAMA3
LAMB3
LAMC2

Figure S6. Co-localization in Xenium data.

(a) Major cell annotations of Xenium data. (b-d) Cell subtype annotations of Epithelial cells, Mes cells, and Fibroblasts. (e) *ITGA3* and *ITGB4* expression distribution in Mes cells. (f-g) Mes and Fibroblast subtype in situ distribution. (h) Left and Middle, Kernel density plots displaying the Fibroblast distribution along X and Y axis. Right, Stacked bar plots showing the proportion of Fibroblasts. P values by Mann-Kendall trend test. (i) EMT-*early* and corresponding ligands in situ distribution. (j) Co-localization of $ITGB4^+$/$ITGA3^+$EMT-*early* with their autocrine ligands.

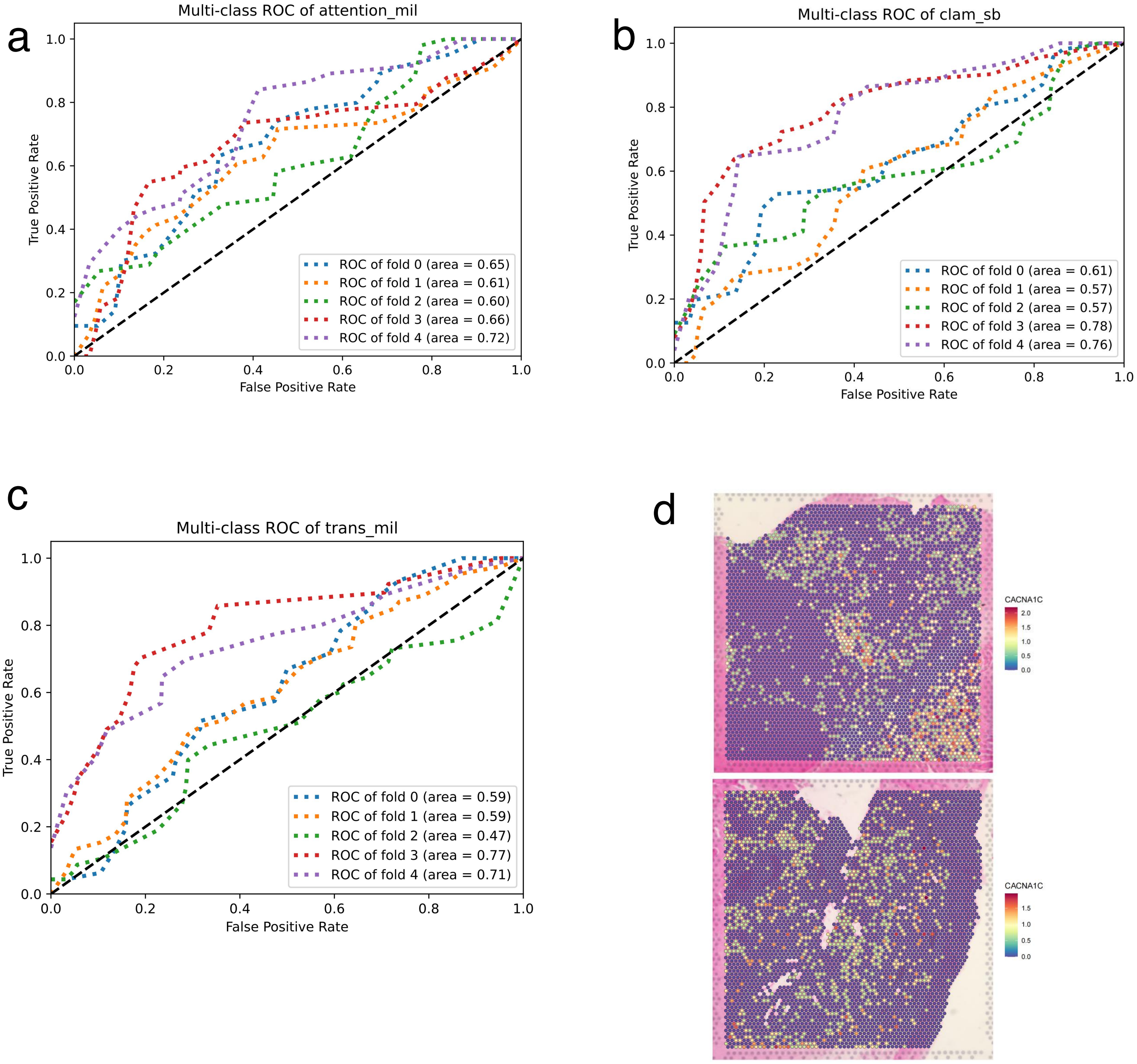
a
Multi-class ROC of attention_mil
True Positive Rate
False Positive Rate
ROC of fold 0 (area = 0.65)
ROC of fold 1 (area = 0.61)
ROC of fold 2 (area = 0.60)
ROC of fold 3 (area = 0.66)
ROC of fold 4 (area = 0.72)
b
Multi-class ROC of clam_sb
True Positive Rate
False Positive Rate
ROC of fold 0 (area = 0.61)
ROC of fold 1 (area = 0.57)
ROC of fold 2 (area = 0.57)
ROC of fold 3 (area = 0.78)
ROC of fold 4 (area = 0.76)
c
Multi-class ROC of trans_mil
True Positive Rate
False Positive Rate
ROC of fold 0 (area = 0.59)
ROC of fold 1 (area = 0.59)
ROC of fold 2 (area = 0.47)
ROC of fold 3 (area = 0.77)
ROC of fold 4 (area = 0.71)
d
CACNA1C
CACNA1C

Figure S7. AMIL model performance evaluation.
(a-c) Five-fold cross-validation performance of the AMIL models. (d) In situ expression patterns of CACNA1C in Visium data.
